# Absence of Visual Input Results in the Disruption of Grid Cell Firing in the Mouse

**DOI:** 10.1016/j.cub.2016.06.043

**Published:** 2016-09-12

**Authors:** Guifen Chen, Daniel Manson, Francesca Cacucci, Thomas Joseph Wills

**Affiliations:** 1 Department of Neuroscience, Physiology, and Pharmacology, UCL, Gower Street, London WC1E 6BT, UK; 2 Department of Cell and Developmental Biology, UCL, Gower Street, London WC1E 6BT, UK; 3 Centre for Mathematics and Physics in the Life Sciences and Experimental Biology, UCL, Gower Place, London WC1E 6BT, UK

## Abstract

Grid cells are spatially modulated neurons within the medial entorhinal cortex whose firing fields are arranged at the vertices of tessellating equilateral triangles [1]. The exquisite periodicity of their firing has led to the suggestion that they represent a path integration signal, tracking the organism’s position by integrating speed and direction of movement [2, 3, 4, 5, 6, 7, 8, 9, 10]. External sensory inputs are required to reset any errors that the path integrator would inevitably accumulate. Here we probe the nature of the external sensory inputs required to sustain grid firing, by recording grid cells as mice explore familiar environments in complete darkness. The absence of visual cues results in a significant disruption of grid cell firing patterns, even when the quality of the directional information provided by head direction cells is largely preserved. Darkness alters the expression of velocity signaling within the entorhinal cortex, with changes evident in grid cell firing rate and the local field potential theta frequency. Short-term (<1.5 s) spike timing relationships between grid cell pairs are preserved in the dark, indicating that network patterns of excitatory and inhibitory coupling between grid cells exist independently of visual input and of spatially periodic firing. However, we find no evidence of preserved hexagonal symmetry in the spatial firing of single grid cells at comparable short timescales. Taken together, these results demonstrate that visual input is required to sustain grid cell periodicity and stability in mice and suggest that grid cells in mice cannot perform accurate path integration in the absence of reliable visual cues.

## Highlights

• Grid cell firing patterns are disrupted in darkness in the mouse
• Grid cells are disrupted even when head direction cell signaling is preserved
• Absence of visual input alters movement velocity modulation of theta frequency
• Temporal firing relationships between grid cell pairs are preserved in the dark

## Teaser

Chen et al. show in the mouse that lack of visual input results in severe disruption of grid cell firing, and that this is not caused by instability in the head direction signal but may be due to changed velocity signaling. Temporal firing relationships between grid cells are preserved, even when their spatial firing patterns are disrupted.

## Results

**Figure 1:**
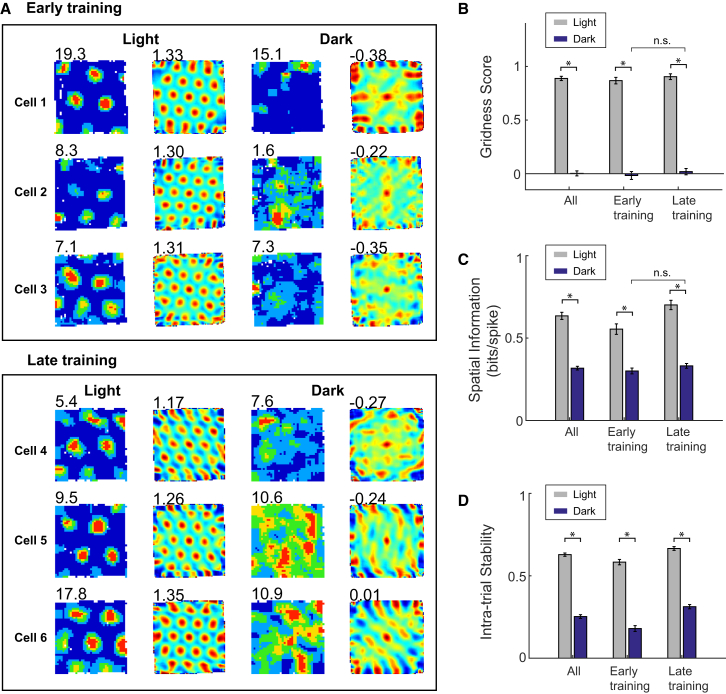
Disruption of Grid Patterns in Complete Darkness (A) Rate maps (left) and spatial auto-correlograms (right) for six representative grid cells simultaneously recorded in a 60 cm square The top three cells (early training) were recorded during the second exposure to the environment in the dark; the bottom three cells (late training) were recorded during the fifth exposure. The leftmost two columns show data from baseline trials in light; the rightmost two columns show trials in darkness. Numbers at the upper left of firing-rate maps are peak firing rate (Hz); those at the upper left of the auto-correlograms are gridness values. (B–D) Comparisons of firing properties of grid cells between light trials (gray) and dark trials (blue): gridness (B), spatial information (C), and intra-trial stability (D). Each bar chart shows the mean values (±SEM) for all recorded grid cells (left group of bars), those recorded during days 1–3 of exposure to darkness (middle group), and those recorded during days 4–9 of exposure to darkness (right group). ^∗^p < 0.001 level; n.s., p > 0.05. See also Figure S1.

In order to determine the importance of visual cues in supporting grid cell firing, we recorded 277 grid cells from the medial entorhinal cortex after mice were introduced into a familiar environment in total darkness. In the absence of visual cues, the characteristic periodicity of grid cells was disrupted (dark condition; Figure 1A) and gridness scores were considerably reduced compared to the baseline light trials (Figures 1B and S1A; 2 × 2 ANOVA, main effect of light condition, N = 277, F_1,275_ = 954.22, p < 0.001). Spatial information and intra-trial stability also decreased significantly in darkness (Figures 1C, 1D, S1B, and S1C; 2 × 2 ANOVA, main effect of light condition: spatial information, F_1,275_ = 372.94, p < 0.001; intra-trial stability, F_1,275_ = 800.75, p < 0.001). Repeated exposures to the familiar environment in the dark did not rescue the deficit, with the disruption in grid firing patterns persisting even after four or more exposures to the familiar environment in the dark (Figures 1B and S1A; 2 × 2 ANOVA, interaction light × experience, F_1,275_ = 0.001, p = 0.991). Similarly, spatial information scores in darkness did not improve upon repeated exposures to the dark condition, despite increases in baseline spatial information scores in the light (Figures 1C and S1B; 2 × 2 ANOVA experience, F_1,275_ = 8.8, p = 0.03; light × experience, F_1,275_ = 12.8, p < 0.001; simple main effects [SME] experience_(light)_, p = 0.001; SME experience_(dark)_, p = 0.167); while intra-trial stability increased slightly after repeated exposures, in both light and dark (Figures 1D and S1C; 2 × 2 ANOVA experience, F_1,275_ = 47.7, p < 0.001; light × experience, F_1,275_ = 3.49, p = 0.063). These results are robust to controlling for the resampling of neurons across days (see Figures S1D–S1F and Supplemental Experimental Procedures for details), and the position and speed sampling did not differ between light and dark, excluding these as potential sources of bias (see Figures S1L–S1N). A subset of grid cells retained above-chance gridness in the dark (defined as a gridness above the 95% confidence-level threshold used to define neurons as grid cells; see Figures S1A and S1G–S1K and Experimental Procedures), but even these grid cells nevertheless showed significant reductions in gridness, spatial information, and spatial stability in darkness (Figure S1J).

To establish whether eliminating visual input disrupts grid firing even when a continuous stream of self-motion information is available, we introduced mice to the familiar environment with the lights on and turned the lights off 10 min after the start of the trial (light-dark condition; Figures S1O–S1Y). Overall, there was no significant improvement over the dark condition: gridness was still significantly lower during the dark phase of the trial compared to the light phase (Figure S1Q; 2 × 2 ANOVA, main effect of light condition, F_1,346_ = 835.33, p < 0.001). However, after several exposures to the light-dark condition (n ≥ 4 exposures), gridness values slightly improved, with some regularity appearing in the firing-rate maps (light × experience, F_1, 346_ = 37.3, p < 0.001; SME experience_(dark)_, p = 0.006; see Figures S1Q–S1S for further quantification).

**Figure 2:**
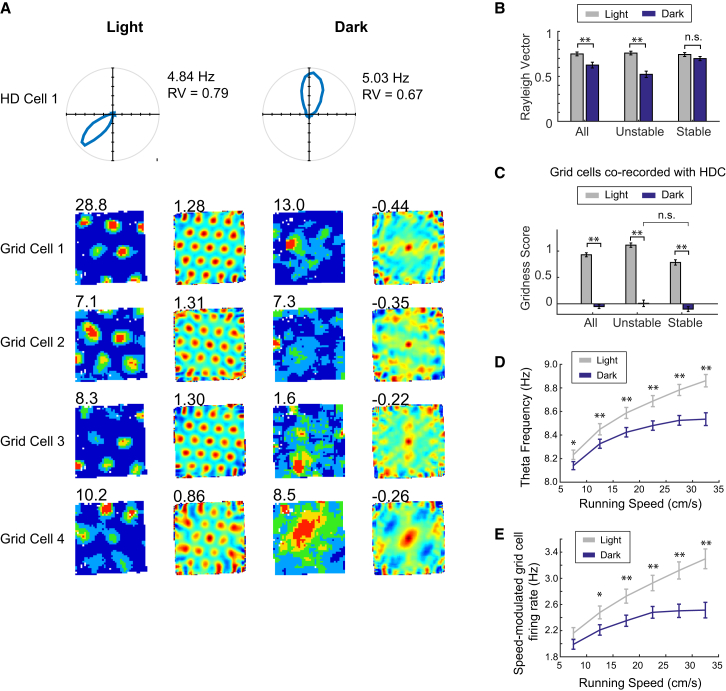
Disruption of Grid Patterns in Darkness Is Accompanied by Stable Directional Input but Altered Velocity Signaling (A) Polar plots for a representative head direction cell showing directional tuning in darkness and firing-rate maps and auto-correlograms for simultaneously recorded grid cells in light trials (left columns) and dark trials (right columns). Numbers at the upper right of polar plots are peak firing rate (Hz) and Rayleigh vector (RV), those at the upper left of firing-rate maps are peak firing rate (Hz), and those at the upper left of auto-correlograms are gridness values. (B) Mean (±SEM) RV scores of all recorded HD cells (n = 34), HD cells that are unstable in the dark (n = 12), and those that are stable in the dark (n = 22). (C) Mean (±SEM) gridness scores of grid cells co-recorded with unstable HD cells and stable HD cells in light (gray) and dark (blue). (D) Relationship between running speed and instantaneous theta frequency of entorhinal cortex local field potential in light (gray) and dark (blue). Lines show the mean (±SEM) instantaneous frequency in each running speed bin (5 cm/s to 35 cm/s in 5 cm/s bins). (E) Relationship between running speed and firing rates of speed-modulated grid cells in light (gray) and dark (blue). Lines show the mean (±SEM) firing rate of all speed-modulated grid cells in each running speed bin. ^∗∗^p < 0.001; ^∗^p < 0.05; n.s., p > 0.05; post hoc differences between light and dark for each speed bin. See also Figure S2.

A stable directional heading signal from the head direction cell circuit is necessary for generating and maintaining grid cells’ regular firing [11], and there is evidence that darkness can induce head direction instability in mice [12]. In order to test whether grid cell disruption in the dark is caused by directional instability, we obtained simultaneous recordings of head direction and grid cells (number of HD cells = 34, number of ensembles with simultaneously recorded HD and grid cells = 17; see Figure 2A for examples). As a measure of head direction (HD) cell stability within a trial, we computed the Rayleigh vector (RV) score, with cells considered to exhibit stable directional tuning in the dark if the ΔRV_light-dark_ fell within one standard deviation of the RV scores for the whole HD cell population during the light trial (ΔRV_light-dark_ ≤ 0.12). Of the 34 HD cells recorded, 22 cells (77%) were classified as stable-HD (with 12/17 [70%] grid cell/HD cell ensembles containing at least one stable-HD; see Figures S2A and S2C). These stable-HDs continued to show strong directional tuning in the absence of visual inputs (Figure 2B; 2 × 2 ANOVA light × HD stability, RV: main effect of light, F_1,32_ = 93.0, p < 0.001; HD stability F_1,32_ = 7.6, p = 0.009; light ×HD stability, F_1,32_ = 48.4, p < 0.001; SME HD stability_(light)_ p = 0.76; SME HD stability_(dark)_ p < 0.001). In some cases, stable-HDs showed the same preferred firing direction in darkness as those in the light session (with 4/12 ensembles displaying an average preferred direction shift < 25°; see Figures S2B and S2C; note that in all cases, pairs of simultaneously recorded HD cells kept consistent preferred direction offsets; see Figure S2B). Grid cells simultaneously recorded with at least one stable-HD showed the same degree of disruption in the dark (with a trend toward greater disruption) as those recorded with unstable-HDs (Figure 2C; n = 88 grid cells; 2 × 2 ANOVA light × HD stability, gridness: main effect of light, F_1,86_ = 321, p < 0.001; HD stability F_1,86_ = 21.0, p < 0.001; light × HD stability, F_1,86_ = 3.70, p = 0.043; SME stability_(dark)_ p = 0.076). These results are robust to controlling for the resampling of neurons across days (see Figures S2D and S2E). These data show that, in the mouse, absence of visual input results in a larger degree of disruption to regular grid cell firing patterns than to HD signaling.

We also co-recorded with grid cells a small number of putative boundary-responsive (BR) cells [13, 14] (n = 8). Interestingly, we observed a qualitatively similar degree of disruption of BR cells’ firing to that of grid cells, with both spatial information and within-trial stability scores of BR cells dropping in the dark (see Figures S2F and S2G).

As we discounted heading instability as the sole source of grid cell disruption, we sought to identify other potential sources of positional error. The two main classes of grid cell model (continuous attractor and oscillatory interference) both include a velocity signal that allows the static grid cell representation to be generated and updated on the basis of the organism’s displacement [2, 3, 4, 5, 6, 7, 8, 9, 10]. Velocity information may be carried, in the hippocampus, by the frequency of theta, the principal oscillation observed in the local field potential during movement (with theta frequency increasing monotonically with running speed [15, 16]). We found a reduction in the increase of theta frequency with running speed in the dark (Figure 2D; 2 × 6 ANOVA light × speed; main effect of speed, F_5,216_ = 22.4, p < 0.001; light, F_1,216_ = 334, p < 0.001; light × speed F_5,216_ = 8.13, p < 0.001). Theta frequency in light was significantly greater than frequency in the dark at all speed values (SME light-dark p < 0.05 for all). However, although reduced, speed modulation of frequency was still present in darkness, as shown by significant post hoc differences between frequency in the speed bins 32.5 cm/s and 17.5 cm/s (SME_dark_; p = 0.042), or between 32.5 cm/s and 12.5 cm/s or slower (p < 0.001). Another potential source of velocity signal to the grid cell network is the speed modulation of firing rate of entorhinal cortex neurons, both “speed cells” [17] and/or speed-modulated grid cells [18, 19]. 160 grid cells (58%) were classified as speed-modulated (see Experimental Procedures). The speed modulation of these cells’ firing was altered in the dark: the degree of firing-rate increase with speed was reduced, and firing rate in the dark was significantly less than that in the light from speeds of greater than 10 cm/s onward (Figure 2E; 2 × 6 ANOVA speed × light: main effect of speed, F_5,954_ = 11.8, p < 0.001; light, F_1,954_ = 100.0, p < 0.001; light × speed F_5,903_ = 4.32, p = 0.001; SME light significant for all speed bins ≥ 12.5 cm/s, p < 0.05). As for frequency, significant differences in rate were present between speed bins 32.5 cm/s and 12.5 cm/s (SME dark; p = 0.021) even in darkness, showing that speed modulation is reduced, but not eliminated, in the dark. Taken together, these results indicate that the animal’s estimation of speed is likely altered by the absence of visual input. It is possible that, similarly to what is found under passive transport conditions [20], the combination of relatively accurate computation of angular displacement (conveyed by the only mildly altered HD signaling) and more significantly altered computation of linear displacement (due to the reduction of theta frequency and grid cell firing dependency on running speed) may be the cause of grid cell disruption in the dark in the mouse.

**Figure 3:**
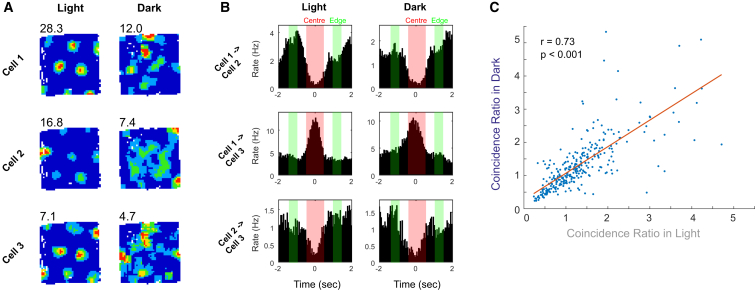
Preserved Temporal Cross-correlations between Grid Cell Pairs in Darkness (A) Rate maps of three representative grid cells in light (left column) and in darkness (right column). (B) Example temporal cross-correlograms between each pairing of the three cells shown (A) in light (left column) and in darkness (right column). The time windows used to calculate the coincidence ratio of the cross-correlogram are shown as transparent colored boxes (center in red; edge in green). (C) The coincidence ratios of all simultaneously recorded grid cell pairs in darkness were significantly correlated with those in light (r = 0.73, p < 0.001). See also Figure S3.

Several models of grid cell firing posit that grid cells are arranged as a low-dimensional continuous attractor [6, 7, 8, 10]. This network structure implies that excitatory/inhibitory relationships between grid cell pairs are fixed and should be preserved even if the network becomes decoupled from external sensory inputs [21, 22]. To assess whether grid cells showed coincident firing (indicative of excitatory coupling) or offset firing (indicative of inhibitory coupling), we constructed temporal cross-correlograms of grid cell pairs’ spike trains and calculated the “coincidence ratio” between the mean rate within the central (±0.5 s) and offset (±1–1.5 s) portions of the correlogram (see Figures 3A and 3B for examples; central areas are marked in red and offset areas in green). Coincidence ratios in the light and dark are highly correlated across all cell pairs (Figure 3C; r = 0.73, p < 0.001), and this strong relationship holds true also when data are analyzed separately for each simultaneously recorded grid cell ensemble (see Figure S3). Co-recorded grid cell pairs therefore show preserved temporal firing relationships even when, in the dark, grid cell representations have lost their spatial stability across the whole trial length.

**Figure 4:**
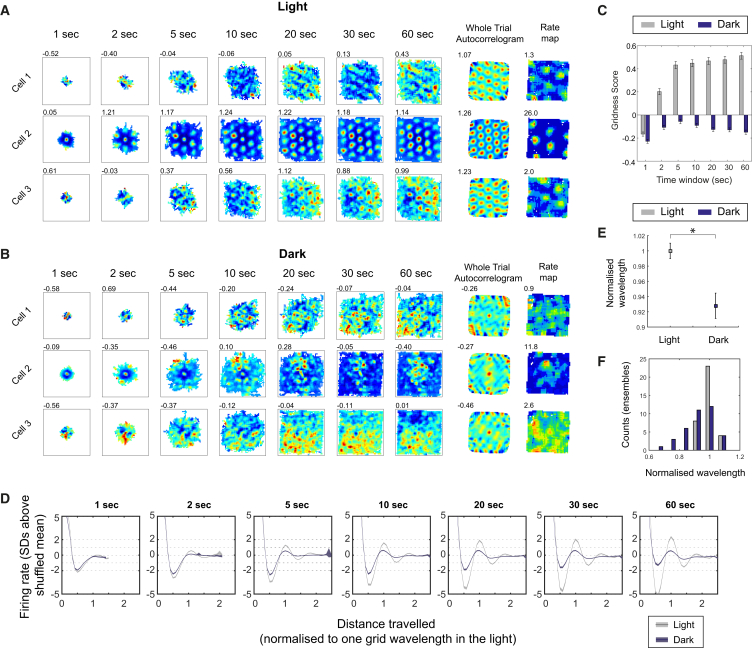
Loss of Hexagonal Symmetry and Compressed Displacement Scale in Grid Cell Firing at Short Timescales in Total Darkness (A) 2D time-windowed spatial displacement firing-rate maps (1, 2, 5, 10, 20, 30, and 60 s windows; first seven columns) for three representative simultaneously recorded grid cells in light, and respective whole-trial spatial auto-correlograms (eighth column) and firing-rate maps (ninth column). For complete ensemble, see Figures S4A and S4B. (B) Same set of cells as in (A), recorded in darkness. (C) Mean (±SEM) gridness scores calculated from the time-windowed maps for time windows of increasing duration, in both light (gray) and dark (blue). (D) 1D time-windowed spatial displacement firing-rate maps (x and y dimensions collapsed to Pythagorean displacement; 1, 2, 5, 10, 20, 30, and 60 s windows) averaged across all grid cells for light (gray) and dark (blue) conditions. The x axis for each plot shows displacement distance, normalized to the grid wavelength in the light for each cell. The y axes show firing rate expressed as SD above the mean of a spatially shuffled control (see Supplemental Experimental Procedures). Shaded areas around the lines show the SEM at each spatial bin. (E) Mean (±SEM) of the wavelengths of time-windowed distance maps (means for each simultaneously recorded ensemble) in both light and dark. Wavelength was defined as the distance to the first peak of the distance map after zero. ^∗^p ≤ 0.01. Data here are based on a 20 s time window. (F) Histogram showing the raw data contributing to the means in (E), i.e., the distributions of ensemble mean wavelengths in light (gray) and dark (blue). See also Figure S4.

The temporal cross-correlogram analyses show that temporal grid cell coupling is preserved at short timescales (on the order of ± 1.5 s). We therefore sought to probe whether the spatial structure of each grid cell’s firing is preserved at comparable timescales, by computing time-windowed spatial displacement firing-rate maps of individual grid cells for both light and dark trials (similarly to [23]). We found no evidence of preserved hexagonal symmetry at timescales ranging from 1 to 60 s (Figures 4A–4C). To test whether grid cells continued to code for distance traveled at short timescales in the dark, we re-computed the time-windowed spatial displacement firing-rate maps, collapsing the x and y dimensions into a single “displacement distance” metric (1D displacement firing-rate maps; see Figure 4D and Supplemental Experimental Procedures for details). We found that a degraded signal for distance traveled was preserved at short timescales in the dark (Figure 4D). Notably, the scale of the distance code (as measured by the distance from the origin to the first peak of the 1D displacement map) was compressed, such that overall the distance between firing peaks was approximately 7% shorter in the dark, at a time window of 20 s (see Figures 4E and 4F; paired t test: t_(36)_ = 3.76, p = 0.001). The displacement scale was also significantly smaller in the dark at time windows of 10 s (96.4% of normalized wavelength, t_(36)_ = 2.25, p = 0.031) and 30 s (94.2% of normalized wavelength, t_(36)_ = 2.21, p = 0.033), but not at 1 s, 2 s, 5 s, or 60 s (data not shown).

## Discussion

Grid cells are widely thought to integrate self-motion information and to provide the rest of the brain with a path integrative input that can be used to calculate one’s location in space even when external sensory input is lacking or too noisy [24, 25]. This view is supported by the finding in the rat that grid cell patterns are preserved when grid cells are recorded in darkness [1, 26]. Surprisingly, we show here that when grid cells are recorded in the mouse in the dark, under very similar conditions to the studies reported above, grid cell firing is severely disrupted (Figure 1). Turning off the lights while the mouse is in the arena (Figures S1O–S1Y) allows a partial rescue of this disruption, but only after several training trials in darkness, indicating that with sufficient training, animals might learn strategies to remain oriented (possibly exploiting olfactory traces left during the initial, illuminated, part of the trial). These data indicate that in mice, grid cells may not always be able to provide an accurate estimate of position solely on the basis of self-motion cues, suggesting that homing abilities in darkness in the mouse (of the kind tested by [27]) might not require stable and periodic grid cell firing. These results are at odds with those obtained in the rat [1, 26] and raise interesting questions as to the source (or sources) of this striking inter-species difference in the resilience of grid signaling to removal of visual cues.

Importantly, we have excluded heading disorientation as the sole and/or critical cause of grid cell disruption. The degree of disruption to the HD signal is not qualitatively comparable to that observed in either grid or border cells. Most HD cells (77%) were stable in the dark. Importantly, even when the HD preferred direction did not change between the light and dark trials, grid cell firing was disrupted and grid symmetry lost in the dark. Such an uncoupling between grid and head direction cells has recently been described in the rat, with passive transportation also selectively affecting grid cell firing patterns while largely sparing head direction firing [20].

The discovery of the grid cell phenomenon and the regularity of grid cells’ firing has inspired a large number of modeling efforts [2, 3, 4, 5, 6, 7, 8, 9, 10, 28], which all share the central idea that grid cells must receive a velocity signal in order to allow the spatial location to be updated on the basis of the animal’s displacement. The source of this velocity signal has been posited to be either the frequency of the theta oscillation or the firing rate of grid cells and speed cells (the three increase linearly with running speed [15, 16, 17, 18, 19]). Interestingly, we have demonstrated here that the relationships between running speed and both theta frequency (Figure 2D) and the firing rate of speed-modulated grid cells (Figure 2E) are both altered when mice explore the familiar environment in the dark. We therefore speculate that optic flow might be an important determinant in the computation of running speed, and that vestibular and proprioceptive information are therefore not sufficient in the mouse to provide an accurate estimate of linear speed (and consequently spatial displacement). This is consistent with a modeling study [29] that demonstrated how optic flow can be used to compute a velocity signal capable of sustaining grid cell firing (see also [30]), and with recent experimental evidence demonstrating that loss of theta frequency and/or firing-rate modulation by speed is associated with disruption of grid signaling [20, 31].

In sum, we observed that darkness may produce small decreases in directional signaling specificity, larger decreases in border cell stability and spatial tuning, and altered signaling of velocity. Future modeling efforts may be useful to establish if and/or how each of these phenomena could contribute to the strong disruption of grid cell firing observed in our study.

The loss of spatial periodicity in grid cell firing in the dark allowed us to probe whether a grid cell network that has lost its stable and regular relationship with external inputs still retains preserved inhibitory and/or excitatory relationships between cell pairs, as postulated by continuous attractor models [6, 7, 8, 10]. Consistent with these models, we found that the temporal relationship (at timescales of 1.5 s) between simultaneously recorded grid cell pairs is spared by darkness (Figures 3 and S3). Our results therefore confirm the findings by Yoon and colleagues [22] showing that under conditions where each grid cell response is deformed, the coupling of grid cell pairs is still preserved, and generalize them to the extreme case where grid cell firing patterns are substantially degraded and no longer bear any obvious spatial relationship to external sensory inputs. Despite preserved temporal coupling between grid cells in the dark, we found no evidence in support of the view that single grid cell firing patterns display hexagonal symmetry at comparatively short timescales, although we found evidence suggesting that, in the dark, a distance code, although compressed, is still present in grid cell firing (Figure 4).

In conclusion, we have demonstrated here that, in the mouse, grid cell firing is reliant on visual input. Absence of visual input results in extensive disruption of grid cell periodicity, possibly by altering running speed computation, as reflected by the reduced dependency of theta frequency and grid cell firing on running speed in the dark. Temporal relationships between grid cell pairs are preserved in the dark, supporting the view that grid cell networks are supported by continuous attractor dynamics, and a degraded code for distance is also preserved. However, we find no evidence that the attractor is able to generate regular hexagonal spatial firing in darkness, even on the shortest of measurable timescales.

## Experimental Procedures

### Subjects and Surgery

Six wild-type mice (C57BL/6J) were implanted with custom-made microdrives, loaded with 17 μm platinum-iridium tetrodes, targeted at the mEC. Tetrodes were implanted 3.0 mm lateral to bregma, 0.2 mm anterior to the transverse sinus, 0.8 mm below the brain surface and angled 4° posteriorly. Electrode position was confirmed postmortem by transcardial perfusion with 4% paraformaldehyde in PBS followed by Nissl staining. All work was performed according to the Animals (Scientific Procedures) Act 1986 and according to Home Office and institutional guidelines.

### Behavioral Training

After recovery from surgery, mice were exposed to the same recording arena every day (20 min per day) to screen for grid cell activity. Electrodes were lowered by 62.5 μm per day until grid cells were found. During all screening and recording sessions, mice foraged for sweetened soy milk drops scattered pseudo-randomly throughout the arena. Trials lasted for 20 min. The floor was cleaned with 70% ethanol between exposures. Experimental sessions began after grid cells were found (after 3–17 screening trials). An experimental session comprised a 20 min trial in the familiar environment in the light and a 20 min trial in the same environment with the lights turned off (lights were turned off before the mouse was placed in the environment). The order of “light” and “dark” trials was counterbalanced across mice and across experience. See Supplemental Experimental Procedures for further details.

### Rate Maps: Assessing Gridness and Spatially Tuned Firing

Spike sorting was performed offline using an automated clustering algorithm (KlustaKwik [32]) followed by a manual review. Firing-rate maps constructed using 1.5 × 1.5 cm spatial bins and a 5 × 5 boxcar filter. Spatial auto-correlograms and “gridness” scores were calculated from the rate maps similarly to [18] (see Supplemental Experimental Procedures for further details). Cells were classified as grid cells if their gridness score in “light” trials exceeded the 95^th^ percentile of a distribution of 1,000 gridness scores derived by spatially temporally shuffling the spike data for that cell. Shuffling was performed similarly to [33](see Supplemental Experimental Procedures for further details). For calculation of intra-trial stability, spatial information, and Rayleigh vector, see Supplemental Experimental Procedures.

### Speed Modulation of Theta Frequency and Grid Cell Firing Rate

#### Theta Frequency

Local-field potential signals recorded concurrently with grid cells were band-pass filtered between 5 and 11 Hz. The Hilbert transform was used to define an instantaneous frequency for each position sample. Position samples were then sorted according to running speed (5 cm/s bins), and the mean frequency for each speed bin was calculated.

#### Firing Rate

Speed-modulated grid cells were defined as in [17]. Following this, for those grid cells that were speed-modulated in either light or dark, the mean firing rate at each speed was defined as the number of spikes occurring at a given speed, divided by the total time during the trial spent moving at that speed. See Supplemental Experimental Procedures for further details.

### Coincidence Ratio and Temporal Cross-correlograms

To compute the “coincidence ratio” from the temporal cross-correlogram, we took the mean of the section with −0.5 s < Δt < +0.5 s and divided by the mean of the two sections with −1.5 s < Δt < −1 s and 1 s < Δt < 1.5 s. The preservation of coincident/non-coincident firing relationships in the dark was then tested using linear regression between the light and dark ratios.

### Time-Windowed Spatial Displacement Firing-Rate Maps

Time-windowed spatial displacement firing-rate maps were constructed following [23]. 1D displacement maps were constructed using the same time-windowing procedure, but following conversation of displacements in the x and y dimensions to Pythagorean distance, before constructing rate maps. For both 1D and 2D maps, to reduce the noise caused by low levels of position sampling, 100 spatially shuffled time-windowed spatial displacement maps were calculated (created by shifting the spike train by a random amount of at least 20 s with respect to the position data), and the real-time-windowed firing-rate values were re-expressed as the number of standard deviations above the mean of the shuffled population for each spatially corresponding bin. Gridness of time-windowed auto-correlograms was assessed using the gridness measure described above, with the exception that the six closest peaks were not defined; rather, the gridness mask derived from the whole-trial auto-correlogram was used instead.

## Author Contributions

G.C., F.C., and T.J.W. designed the experiments and analyses. G.C. collected the data. G.C., D.M., F.C., and T.J.W. analyzed the data. G.C., F.C., and T.J.W. contributed to drafting the manuscript.

## Supplemental Information

Document S1. Figures S1–S4 and Supplemental Experimental Procedures

Document S2. Article plus Supplemental Information

## References

[1] Hafting T., Fyhn M., Molden S., Moser M.B., Moser E.I. (2005). Microstructure of a spatial map in the entorhinal cortex. Nature.

[2] Burgess N., Barry C., O’Keefe J. (2007). An oscillatory interference model of grid cell firing. Hippocampus.

[3] Blair H.T., Gupta K., Zhang K. (2008). Conversion of a phase- to a rate-coded position signal by a three-stage model of theta cells, grid cells, and place cells. Hippocampus.

[4] Hasselmo M.E. (2008). Grid cell mechanisms and function: contributions of entorhinal persistent spiking and phase resetting. Hippocampus.

[5] Welday A.C., Shlifer I.G., Bloom M.L., Zhang K., Blair H.T. (2011). Cosine directional tuning of theta cell burst frequencies: evidence for spatial coding by oscillatory interference. J. Neurosci..

[6] Fuhs M.C., Touretzky D.S. (2006). A spin glass model of path integration in rat medial entorhinal cortex. J. Neurosci..

[7] McNaughton B.L., Battaglia F.P., Jensen O., Moser E.I., Moser M.B. (2006). Path integration and the neural basis of the ‘cognitive map’. Nat. Rev. Neurosci..

[8] Burak Y., Fiete I.R. (2009). Accurate path integration in continuous attractor network models of grid cells. PLoS Comput. Biol..

[9] Bush D., Burgess N. (2014). A hybrid oscillatory interference/continuous attractor network model of grid cell firing. J. Neurosci..

[10] Pastoll H., Solanka L., van Rossum M.C.W., Nolan M.F. (2013). Feedback inhibition enables θ-nested γ oscillations and grid firing fields. Neuron.

[11] Winter S.S., Clark B.J., Taube J.S. (2015). Spatial navigation. Disruption of the head direction cell network impairs the parahippocampal grid cell signal. Science.

[12] Yoder R.M., Taube J.S. (2009). Head direction cell activity in mice: robust directional signal depends on intact otolith organs. J. Neurosci..

[13] Solstad T., Boccara C.N., Kropff E., Moser M.B., Moser E.I. (2008). Representation of geometric borders in the entorhinal cortex. Science.

[14] Lever C., Burton S., Jeewajee A., O’Keefe J., Burgess N. (2009). Boundary vector cells in the subiculum of the hippocampal formation. J. Neurosci..

[15] Sławińska U., Kasicki S. (1998). The frequency of rat’s hippocampal theta rhythm is related to the speed of locomotion. Brain Res..

[16] Jeewajee A., Barry C., O’Keefe J., Burgess N. (2008). Grid cells and theta as oscillatory interference: electrophysiological data from freely moving rats. Hippocampus.

[17] Kropff E., Carmichael J.E., Moser M.-B., Moser E.I. (2015). Speed cells in the medial entorhinal cortex. Nature.

[18] Sargolini F., Fyhn M., Hafting T., McNaughton B.L., Witter M.P., Moser M.B., Moser E.I. (2006). Conjunctive representation of position, direction, and velocity in entorhinal cortex. Science.

[19] Wills T.J., Barry C., Cacucci F. (2012). The abrupt development of adult-like grid cell firing in the medial entorhinal cortex. Front. Neural Circuits.

[20] Winter S.S., Mehlman M.L., Clark B.J., Taube J.S. (2015). Passive Transport Disrupts Grid Signals in the Parahippocampal Cortex. Curr. Biol..

[21] Fyhn M., Hafting T., Treves A., Moser M.B., Moser E.I. (2007). Hippocampal remapping and grid realignment in entorhinal cortex. Nature.

[22] Yoon K., Buice M.A., Barry C., Hayman R., Burgess N., Fiete I.R. (2013). Specific evidence of low-dimensional continuous attractor dynamics in grid cells. Nat. Neurosci..

[23] Bonnevie T., Dunn B., Fyhn M., Hafting T., Derdikman D., Kubie J.L., Roudi Y., Moser E.I., Moser M.B. (2013). Grid cells require excitatory drive from the hippocampus. Nat. Neurosci..

[24] Poucet B., Sargolini F., Song E.Y., Hangya B., Fox S., Muller R.U. (2013). Independence of landmark and self-motion-guided navigation: a different role for grid cells. Philos. Trans. R. Soc. Lond. B Biol. Sci..

[25] Bush D., Barry C., Burgess N. (2014). What do grid cells contribute to place cell firing?. Trends Neurosci..

[26] Barry C., Ginzberg L.L., O’Keefe J., Burgess N. (2012). Grid cell firing patterns signal environmental novelty by expansion. Proc. Natl. Acad. Sci. USA.

[27] Yoder R.M., Goebel E.A., Köppen J.R., Blankenship P.A., Blackwell A.A., Wallace D.G. (2015). Otolithic information is required for homing in the mouse. Hippocampus.

[28] Schmidt-Hieber C., Häusser M. (2013). How to build a grid cell. Philos. Trans. R. Soc. Lond. B Biol. Sci..

[29] Raudies F., Mingolla E., Hasselmo M.E. (2012). Modeling the influence of optic flow on grid cell firing in the absence of other cues1. J. Comput. Neurosci..

[30] Raudies F., Hinman J.R., Hasselmo M.E. (2016). Modelling effects on grid cells of sensory input during self-motion. J. Physiol..

[31] Jacob P.-Y., Poucet B., Liberge M., Save E., Sargolini F. (2014). Vestibular control of entorhinal cortex activity in spatial navigation. Front. Integr. Nuerosci..

[32] Kadir S.N., Goodman D.F.M., Harris K.D. (2014). High-dimensional cluster analysis with the masked EM algorithm. Neural Comput..

[33] Wills T.J., Cacucci F., Burgess N., O’Keefe J. (2010). Development of the hippocampal cognitive map in preweanling rats. Science.

