# Supplementary material for: Absence of Visual Input Results in the Disruption of Grid Cell Firing in the Mouse
Source: Curr Biol. 2016 Sep 12;26(17):2335–42. doi: 10.1016/j.cub.2016.06.043 (PMC5026695; doi:10.1016/j.cub.2016.06.043)
Supplement: Document S1. Figures S1–S4 and Supplemental Experimental Procedures [file mmc1.pdf]

Current Biology, Volume *26*

Supplemental Information

Absence of Visual Input Results in the Disruption of Grid Cell Firing in the Mouse

Guifen Chen, Daniel Manson, Francesca Cacucci, and Thomas Joseph Wills

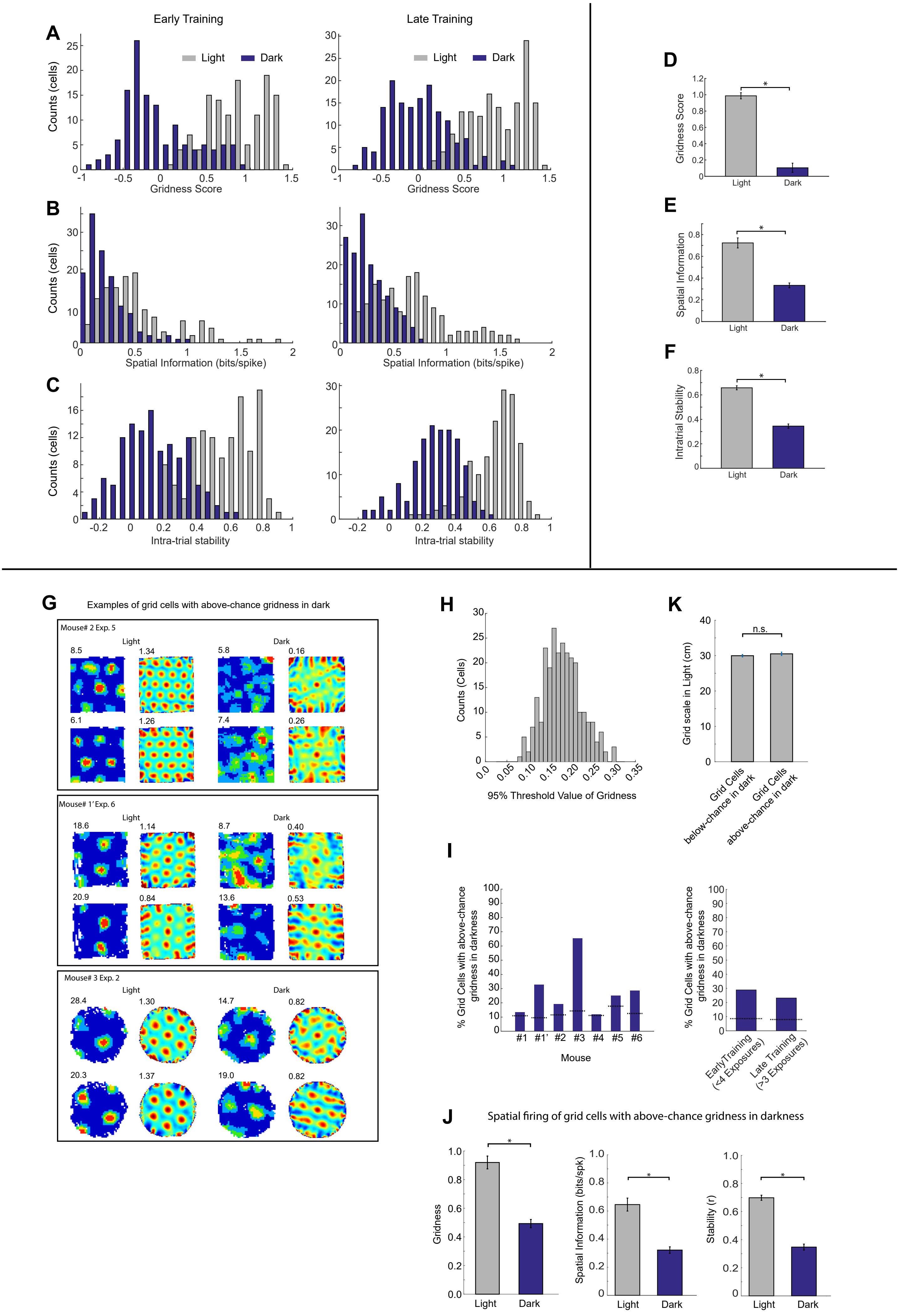

A
Early Training
Late Training
Light
Dark
Counts (cells)
Gridness Score
B
Spatial Information (bits/spike)
C
Intra-trial stability
D
Gridness Score
E
Spatial Information
F
Intratrial Stability
*
G
Examples of grid cells with above-chance gridness in dark
Mouse# 2 Exp. 5
8.5
1.34
5.8
0.16
6.1
1.26
7.4
0.26
Mouse# 1' Exp. 6
18.6
1.14
8.7
0.40
20.9
0.84
13.6
0.53
Mouse# 3 Exp. 2
28.4
1.30
14.7
0.82
20.3
1.37
19.0
0.82
H
Counts (Cells)
95% Threshold Value of Gridness
K
n.s.
Grid scale in Light (cm)
Grid Cells below-chance in dark
Grid Cells above-chance in dark
I
% Grid Cells with above-chance gridness in darkness
#1
#1'
#2
#3
#4
#5
#6
Mouse
EarlyTraining (<4 Exposures)
Late Training (>3 Exposures)
J
Spatial firing of grid cells with above-chance gridness in darkness
Gridness
Spatial Information (bits/spk)
Stability (r)

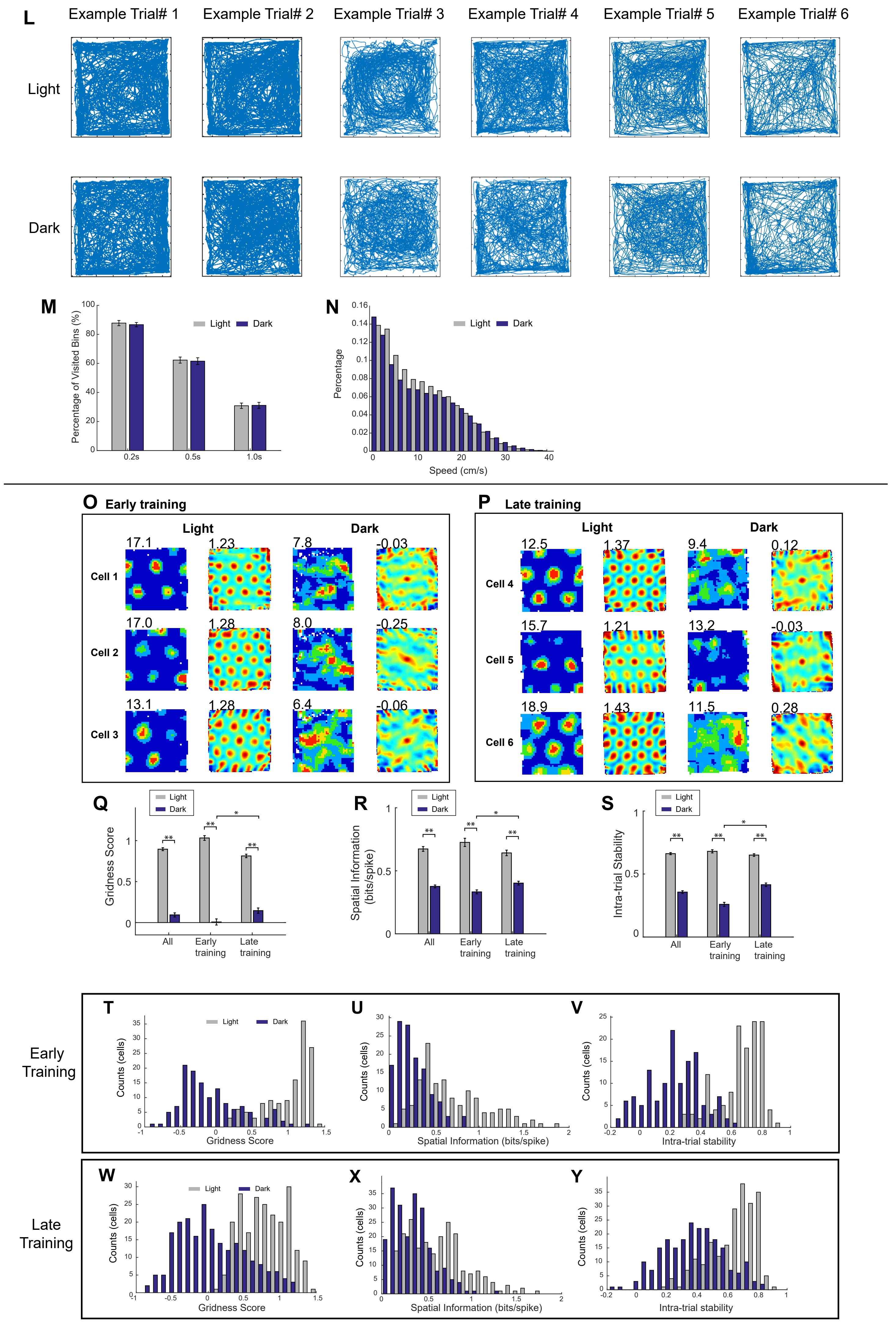
L
Example Trial# 1
Example Trial# 2
Example Trial# 3
Example Trial# 4
Example Trial# 5
Example Trial# 6
Light
Dark
M
Percentage of Visited Bins (%)
Light
Dark
0.2s
0.5s
1.0s
N
Percentage
Light
Dark
Speed (cm/s)
O
Early training
Light
Dark
Cell 1
Cell 2
Cell 3
17.1
1.23
7.8
-0.03
17.0
1.28
8.0
-0.25
13.1
1.28
6.4
-0.06
P
Late training
Light
Dark
Cell 4
Cell 5
Cell 6
12.5
1.37
9.4
0.12
15.7
1.21
13.2
-0.03
18.9
1.43
11.5
0.28
Q
Gridness Score
Light
Dark
All
Early training
Late training
R
Spatial Information (bits/spike)
S
Intra-trial Stability
T
U
V
Early Training
Counts (cells)
Gridness Score
Spatial Information (bits/spike)
Intra-trial stability
W
X
Y
Late Training

Figure S1, related to main figure 1. Grid cell firing is disrupted by the absence of visual input in the mouse. (A-C) Histograms displaying distributions of Gridness (A), Spatial information (B) and Intra-trial Stability (C) for all Grid cells recorded in the light (grey bars) and dark (blue bars) conditions. Data refers to early training phase (leftmost panels; n trials <4) and late training phase (rightmost panels; n trials >=4). Note that all animals had experienced 3-17 trials in the familiar environment in the light before dark training began. **(D-F) Controlling for possible grid cell resampling over experimental days does not alter results.** Mean values (±SEM) for Gridness (D), Spatial information (E) and Intra-trial Stability (F) for a subset of the complete dataset, derived from only one ensemble of co-recorded grid cells per animal (ensemble with largest number of grid cells, total number of grid cells N=72; light condition in grey, dark condition in blue). Paired t-tests demonstrate significant drops in gridness (t_{71} = 14.75; p <0.001), spatial information (t_{71} = 11.53; p <0.001) and intra-trial stability (t_{71} = 15.13; p <0.001) in the dark, replicating results obtained from analyses of complete dataset and thus excluding the possibility that neuronal resampling results in significant bias. Asterisks denote p <0.001 significance level. **(G-K) A subset of grid cells show spatial firing disruption, despite retaining above-chance gridness scores in the darkness.** (G) Firing rate maps and autocorrelograms of example grid cells with above-chance gridness in the dark (defined as gridness in the dark greater than the 95% confidence threshold used to define neurons as grid cells; see Experimental Procedures and figure S1H). Numbers top left of rate maps show peak firing rate, top left of autocorrelograms show gridness score. Left-most two columns show firing in light, right-most two columns firing in darkness. Six examples from three simultaneously recorded ensembles are shown, illustrating low (top), average (middle) and high (bottom) gridness scores in the dark. See figure S1A for further information regarding where these examples sit in comparison to the population of all gridness scores in the dark. (H) Distribution of the 95% confidence thresholds used to define neurons as grid cells in the light, for all cells defined as grid cells (one threshold per cell, see Experimental Procedures). The mean (± SEM) threshold level was 0.18 (±0.04). All analyses in (I-K) relate to those cells whose gridness score in the dark remained above the 95% confidence threshold derived from that cell's firing in the light (69/277 grid cells). (I) Percentages of grid cells with above-chance gridness in the dark, for data grouped by mouse (left panel) and by experience in darkness (right panel). Black dashed lines show the 95% confidence level for the percentages of above-chance cells expected from spatially random firing in the dark (as spatially random data will score higher than threshold 5% of times). Above-chance gridness in darkness does not appear systematically distributed, with the exception of one mouse (mouse #3) which showed substantial preservation of grid cell firing in darkness. (J) Means and SEMs of gridness (left panel), spatial information (middle panel) and spatial stability (right panel), in light and dark, for grid cells with above-chance gridness in darkness. All three scores show a significant reduction in darkness (paired t-tests): gridness, t(68)=8.66, p<0.001; spatial information, t(68)=9.34, p<0.001; stability, t(68)=13.6, p<0.001. (K) Grid cells with gridness values in the dark which are above or below the 95% confidence level of the shuffled maps do not have significantly different grid scales in the light (t-test: t(275)=0.86, p=0.39). **(L-N) Position and speed sampling are similar in the light and dark conditions.** (L) Twelve representative examples of paths taken by mice in the light (top plots) and related dark (bottom plots) trials, demonstrating even coverage of position across the whole environment in both experimental conditions. (M) Mean values (±SEM) of the percentage of visited bins with occupancy values of at least 0.2 secs (leftmost bars), 0.5 secs (middle set of bars) and 1.0 secs (rightmost bars), during the light (grey bars) and dark (blue) conditions. No differences between dark and light occupancy are evident (paired t-tests, 0.2s occupancy: t(38) = -0.70, p = 0.48; 0.5s occupancy: t(38) = -0.55, p = 0.58; 10s occupancy: t(38) = 0.32, p = 0.74). (N) Histograms displaying distribution of running speed in the light (grey bars) and the dark (blue bars). Overall distributions are similar. Mice on average run slightly faster in the dark (mean speed in light: 10.29cm/s ±0.25; mean speed in dark: 11.13 cm/sec ±0.32; Wilcoxon rank test: p=0.047). **(O-Y) Disruption of grid patterns when visual cues are removed during exploration.** (O-P) Firing rate maps and spatial auto-correlograms for 6 representative grid cells simultaneously recorded in a 60 cm square. Three cells in the right box ('Early Training') were recorded during 2nd exposure to the environment in the dark, 3 cells in the left box ('Late Training'), during 6th exposure. Within boxes, leftmost two columns are from baseline trials in light, and rightmost two columns are from light-dark trials when lights were switched off while animals were exploring the environment (in contrast to the mouse starting exploration in darkness, Figure 1 and S1A-E). Numbers top of firing rate maps are peak firing rate (Hz), those on autocorrelograms are gridness values. (Q-S) Comparisons of firing properties of grid cells between light (grey) and dark trials (blue); gridness (Q), spatial information (R), intra-trial stability (S). Each bar chart shows the mean values (±SEM) for all recorded grid cells (left group of bars), those recorded during days 1-3 of exposure to darkness (middle group of bars) and those recorded during days 4-8 of exposure to darkness (right group of bars). All data were analysed using a 2x2 (light*experience) ANOVA, the resulting statistics are as follows. Gridness: main effect of light $F_{1,346}$=835, p<0.001; experience $F_{1,346}$=1.56, p=0.21; light*experience $F_{1,346}$=37.1, p<0.001. SME experience(dark), p=0.006. Spatial Information: main effect of light $F_{1,346}$=511, p<0.001; experience $F_{1,346}$=0.63, p=0.80; light*experience $F_{1,346}$=30.0, p<0.001. SME experience(dark), p=0.03. Intra-trial stability: main effect of light $F_{1,346}$=847, p<0.001; experience $F_{1,346}$=17.1, p<0.001; light*experience $F_{1,346}$=67.7, p<0.001. SME experience(dark), p=0.03.(T-Y) Histograms displaying distributions of Gridness (T,W), Spatial information (U,X) and Intra-trial Stability (V,Y) for all Grid cells recorded in the light (grey bars) and dark (blue bars) conditions data refers to early training phase (top panels; n trials <4) and late training phase (bottom panels). ** represents significance at p<0.001 level, * represents sigificance at p<0.05.

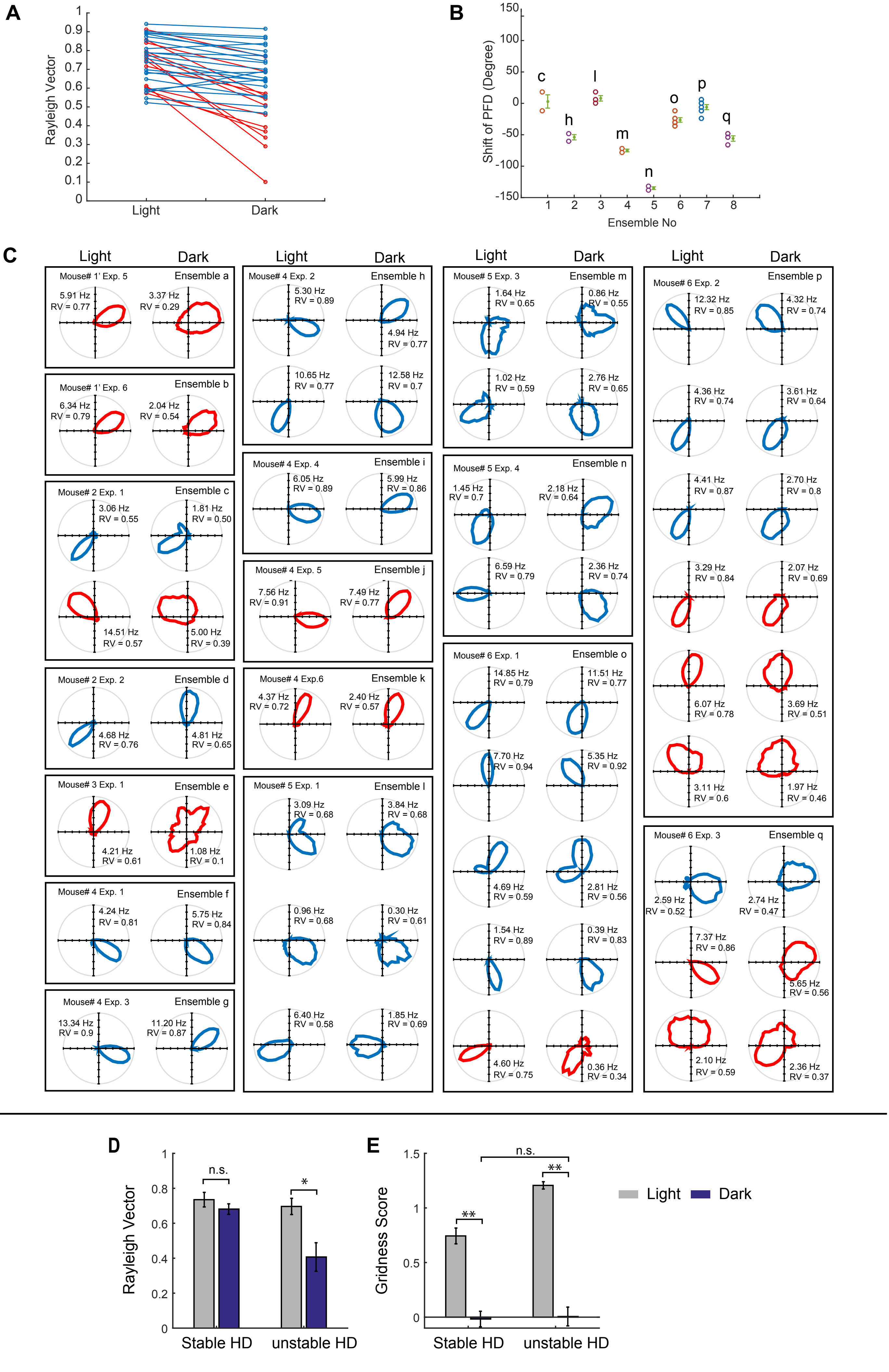

A
Rayleigh Vector
Light
Dark
B
Shift of PFD (Degree)
Ensemble No
c
h
l
m
n
o
p
q
C
Light
Dark
Mouse# 1' Exp. 5
Ensemble a
5.91 Hz
RV = 0.77
3.37 Hz
RV = 0.29
Mouse# 1' Exp. 6
Ensemble b
6.34 Hz
RV = 0.79
2.04 Hz
RV = 0.54
Mouse# 2 Exp. 1
Ensemble c
3.06 Hz
RV = 0.55
1.81 Hz
RV = 0.50
14.51 Hz
RV = 0.57
5.00 Hz
RV = 0.39
Mouse# 2 Exp. 2
Ensemble d
4.68 Hz
RV = 0.76
4.81 Hz
RV = 0.65
Mouse# 3 Exp. 1
Ensemble e
4.21 Hz
RV = 0.61
1.08 Hz
RV = 0.1
Mouse# 4 Exp. 1
Ensemble f
4.24 Hz
RV = 0.81
5.75 Hz
RV = 0.84
Mouse# 4 Exp. 3
Ensemble g
13.34 Hz
RV = 0.9
11.20 Hz
RV = 0.87
Mouse# 4 Exp. 2
Ensemble h
5.30 Hz
RV = 0.89
4.94 Hz
RV = 0.77
10.65 Hz
RV = 0.77
12.58 Hz
RV = 0.7
Mouse# 4 Exp. 4
Ensemble i
6.05 Hz
RV = 0.89
5.99 Hz
RV = 0.86
Mouse# 4 Exp. 5
Ensemble j
7.56 Hz
RV = 0.91
7.49 Hz
RV = 0.77
Mouse# 4 Exp.6
Ensemble k
4.37 Hz
RV = 0.72
2.40 Hz
RV = 0.57
Mouse# 5 Exp. 1
Ensemble l
3.09 Hz
RV = 0.68
3.84 Hz
RV = 0.68
0.96 Hz
RV = 0.68
0.30 Hz
RV = 0.61
6.40 Hz
RV = 0.58
1.85 Hz
RV = 0.69
Mouse# 5 Exp. 3
Ensemble m
1.64 Hz
RV = 0.65
0.86 Hz
RV = 0.55
1.02 Hz
RV = 0.59
2.76 Hz
RV = 0.65
Mouse# 5 Exp. 4
Ensemble n
1.45 Hz
RV = 0.7
2.18 Hz
RV = 0.64
6.59 Hz
RV = 0.79
2.36 Hz
RV = 0.74
Mouse# 6 Exp. 1
Ensemble o
14.85 Hz
RV = 0.79
11.51 Hz
RV = 0.77
7.70 Hz
RV = 0.94
5.35 Hz
RV = 0.92
4.69 Hz
RV = 0.59
2.81 Hz
RV = 0.56
1.54 Hz
RV = 0.89
0.39 Hz
RV = 0.83
4.60 Hz
RV = 0.75
0.36 Hz
RV = 0.34
Mouse# 6 Exp. 2
Ensemble p
12.32 Hz
RV = 0.85
4.32 Hz
RV = 0.74
4.36 Hz
RV = 0.74
3.61 Hz
RV = 0.64
4.41 Hz
RV = 0.87
2.70 Hz
RV = 0.8
3.29 Hz
RV = 0.84
2.07 Hz
RV = 0.69
6.07 Hz
RV = 0.78
3.69 Hz
RV = 0.51
3.11 Hz
RV = 0.6
1.97 Hz
RV = 0.46
Mouse# 6 Exp. 3
Ensemble q
2.59 Hz
RV = 0.52
2.74 Hz
RV = 0.47
7.37 Hz
RV = 0.86
5.65 Hz
RV = 0.56
2.10 Hz
RV = 0.59
2.36 Hz
RV = 0.37
D
Rayleigh Vector
n.s.
*
Stable HD
unstable HD
E
Gridness Score
n.s.
**
**
Stable HD
unstable HD
Light
Dark

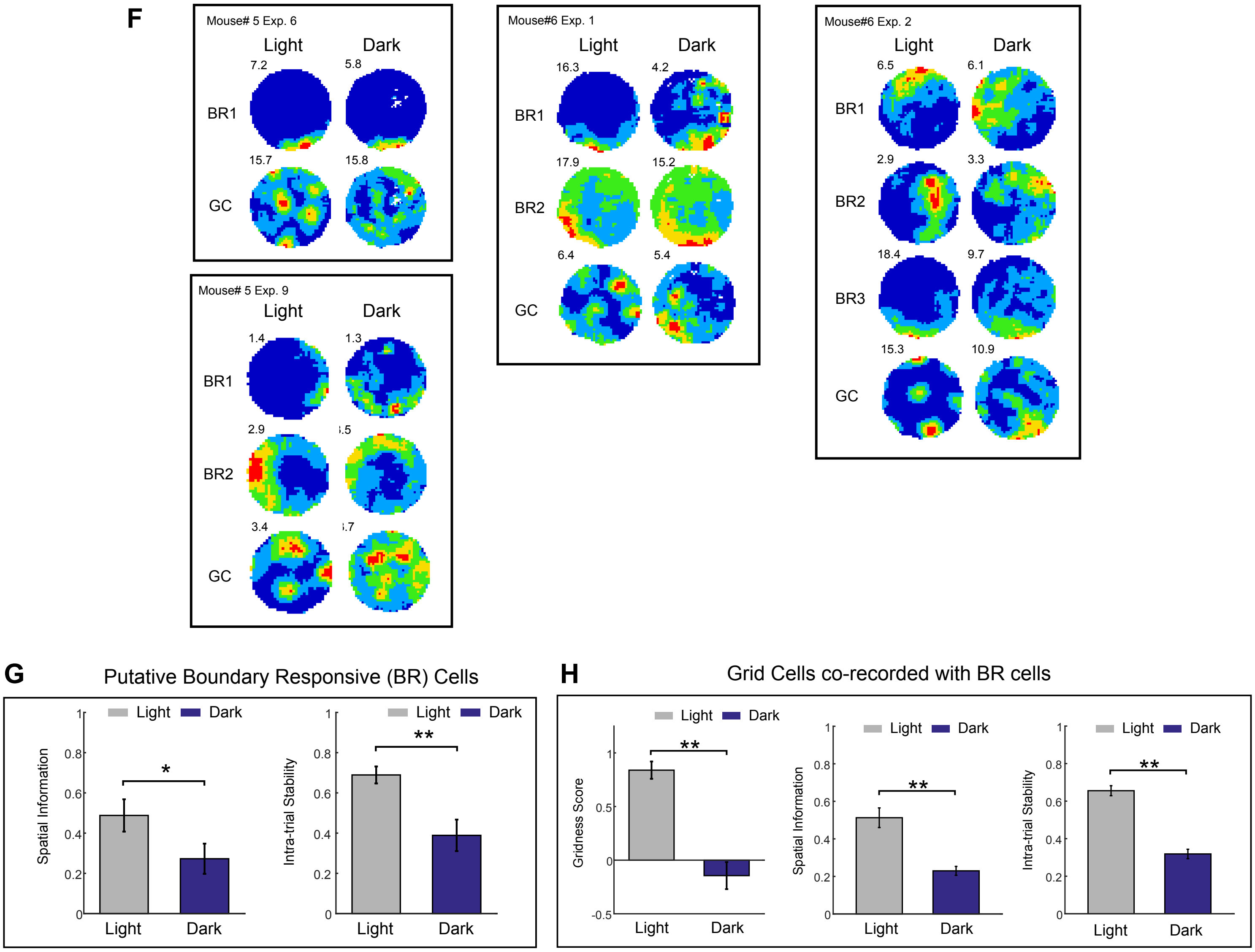

Figure S2, related to main figure 2. (A-C) HD signalling is relatively spared in darkness. (A) Rayleigh Vector (RV) values of all recorded head direction cells (RV>0.5 in light sessions), in both light and dark trials. Lines connect the light and dark values for individual HD cells. Blue lines refer to Stable HD cells ($deltaRV_{light\text{-}dark}$ <=0.12), red to HD cells which become unstable in the dark ($deltaRV_{light\text{-}dark}$ >0.12). (**B**) Co-recorded HD cells retain their relative preferred directions in darkness, regardless of stability. Graph depicting shift in preferred directions between dark and light trials for all HD cells simultaneously recorded with at least another HD cell, either stable or unstable (data refers to all ensembles containing at least 2 HD cells). Data are grouped per ensemble of co-recorded HD cells and letter on top of each vertical group of circles identifies ensemble (related polar plots are displayed in panel S2C). Circles show preferred direction shifts for indivdual HDCs, green lines and error bars show the circular mean and standard deviation of the preferred direction shifts within each ensemble. (C) Polar plots for all Head direction (HD) cells co-recorded with grid cells. For each cell, light trials are shown on the left, dark trials on the right. Black boxes group the 34 individual cells into the 17 simultaneously recorded ensembles from which they are drawn. Number on polar plots are peak firing rate (Hz), and Raleigh Vector (RV) values. Blue polar plots refer to Stable HD cells ($deltaRV_{light\text{-}dark}$ <=0.12), red to HD cells which become unstable in the dark ($deltaRV_{light\text{-}dark}$ >0.12). **(D-E) HD cells' directional tuning is preserved relative to grid firing in the dark, even after controlling for potential resampling of neurons over different experimental days.** Data were analysed from one ensemble per mouse only, that with the greatest number of HD cells (N=15 HD cells, 9 (60%) stable, 6 (30%) unstable). As for the whole dataset, RV values are significantly different between the light and dark condition for unstable HD cells only (panel D; 2x2 ANOVA light*HDC stability, RV: main effect of light, $F_{1,13}$=30.8, p<0.001; HDC stability $F_{1,13}$=6.3, p=0.026; light*HDC stability, $F_{1,13}$=14.5, p=0.002; SME HDC stability(light) p=0.55; SME HDC stability(dark) p=0.03), but co-recorded grid cells (N=49) are equally disrupted whether recorded with stable or unstable HD cells (panel E, 2x2 ANOVA light*HDC stability, gridness: main effect of light, $F_{1,47}$=170, p<0.001; HDC stability $F_{1,47}$=15.0, p<0.001; light*HDC stability, $F_{1,47}$=8.6, p=0.005; SME HDC stability(dark) p=0.84). * denotes significance at p<0.05 level, ** at p<0.001 level. **(F-H) Putative boundary related firing is disrupted in darkness whilst retaining preferential firing near environmental walls.** (F) Firing rate maps for putative boundary responsive cells (BR cells; N=8, top) and examples of co-recorded grid cells (GC cells, bottom) in the light (leftmost maps) and dark (rightmost maps) condition. Black boxes group maps into the 4 simultaneously recorded ensembles from which they are drawn. Numbers top left of firing rate maps indicate peak firing rate (in Hz). (G-H) Comparisons of firing properties of BR cells (G) and grid cells (H) between light (grey) and dark trials (blue). Each bar chart shows the mean values (±SEM) for all recorded BR cells (G) and co-recorded grid cells (H). (G) Both spatial information and intra-trial stability of BR cells are significantly reduced in the dark (paired t-test, spatial information: t(8)=2.84, p=0.022; intra-trial stability: t(8)=4.65, p=0.002). (H) Gridness, spatial information, intra-trial stability of grid cells co-recorded with BR cells all drop significantly in the dark (gridness: t(10)=3.48, p=0.006; spatial information: t(10)=4.61, p=0.001; intra-trial stability: t(10)=4.39, p=0.001).

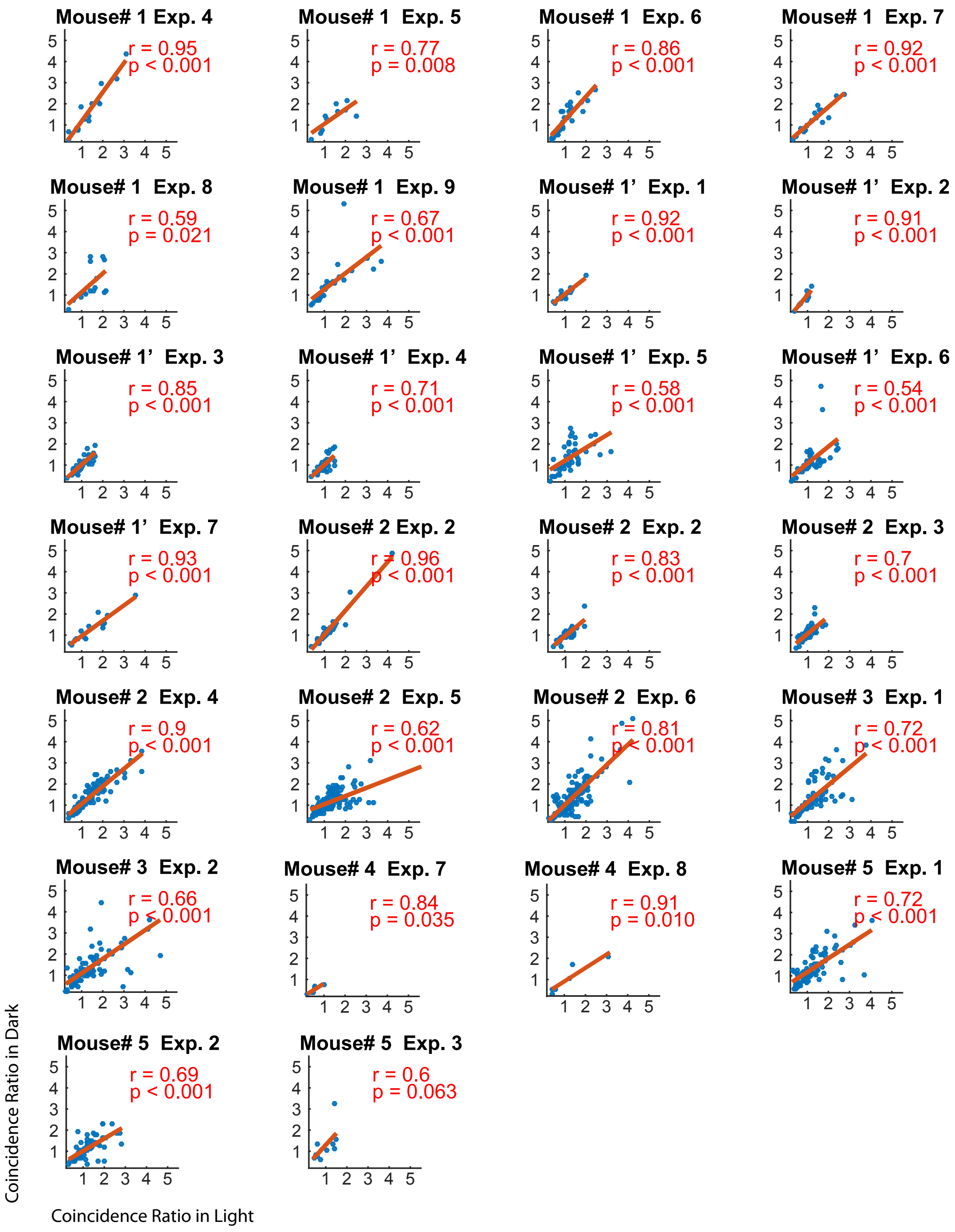

Figure S3, related to main figure 3. Grid cells retain temporal coupling at short timescales (1.5sec) in darkness. Coincidence ratios of temporal cross-correlograms between grid cells pairs in light and darkness, split into simultaneously recorded ensembles. Each plot shows the coincidence ratio of all pairings between a set of simultaneously recorded grid cells, in darkness in comparison to that in light. Only ensembles in which 4 or more grid cells were simultaneously recorded are shown. Numbers in red show the r and p-values for linear regression between the light and dark coincidence ratios.

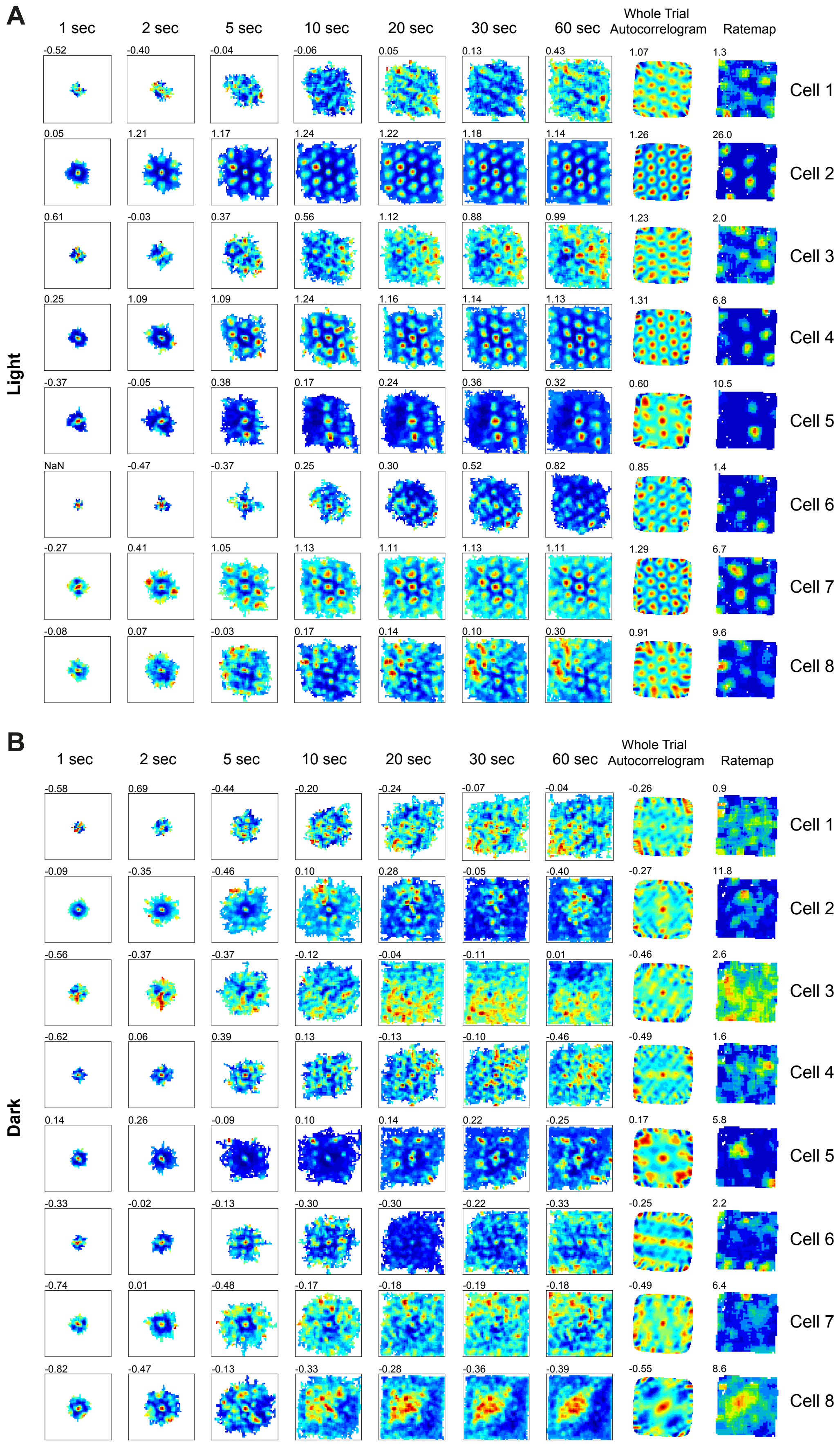

Figure S4, realted to main figure 4. No evidence for hexagonal symmetry in grid cell firing, even at short timescales. (A) 2D Time-windowed spatial displacement firing rate maps (1, 2, 5, 10, 30 and 60 sec windows; five leftmost columns), for a representative example of co-recorded grid cell ensemble in light, and respective whole trial spatial auto-correlograms (the sixth column) and firing rate maps (seventh column) (B) As for (A), the same set of cells recorded in darkness (same ensemble from which the 3 representative examples shown in Figure 4 were drawn).

Supplemental Experimental Procedures

Subjects and surgery. Subjects (6 C57Bl/6 mice) were aged 2-5 months and weighed 23-30 grams at the time of surgery. Mice were housed under 12:12 inverted light-dark cycle, with lights on at 10am. All work was carried out under the Animals (Scientific Procedures) Act 1986 and according to Home Office and institutional guidelines. Mice were implanted with custom-made microdrives targeting the mEC. Each microdrive was loaded with 32 HML-coated 17-µm Platinum-Iridium (90-10%) electrodes twisted into 8 separate tetrodes. Electrodes were electroplated in a Platinum solution to <200kΩ impedance. Following surgery animals were allowed at least one week recovery.

Behavioural Training/Recording environment details. After recovery from surgery, mice were exposed to the same recording arena every day (20 mins day) to screen for grid cell activity. Tetrodes were lowered by 62.5 um each day, until grid cell activity was identified, after which recording sessions began (and tetrodes were no longer moved after this time point). One of three different arenas was used for different mice: (1) a 50 cm diameter circle, (2) a 60 cm diameter circle, (3) a 60x60 cm square. All recording arenas were placed on a black Trespa 'Toplab' surface (Trespa International B.V., Weert, Netherlands) that served as a floor, and surrounded by a circular set of black curtains. A white cue-card (A0, 84 x 119 cm), illuminated by a 40 W lamp, was the only directionally polarising cue within the black curtains. One mouse (mouse #1) underwent the process of being familiarised with the recording arena and then exposed to darkness twice, the second time in a different laboratory and using a different recording arena to those used the first time. Data from exposure in the second laboratory was treated separately to that from the first when quantifying experience, data from mouse #1 that was collected in the second laboratory is designated mouse #1'.

Experimental Protocol. Extra-cellular waveforms were recorded using the "DACQ" recording system (Axona). During recording, the mouse's position and head orientation was tracked (50Hz sampling rate) using two infra-red LEDs attached to the micro-drive at a fixed angle and spacing (5 cm apart). Brief losses of LED data due to cable obstruction were corrected with linear interpolation between known position values. Interpolation was carried out for each LED separately. The position values for each LED were then smoothed, separately, using a 400ms long boxcar filter. Finally, the separate LED positions were used to calculate the averaged position and heading for each position sample. During 'dark' trials, the lights were turned off before the mouse entered into the recording arena, and were not turned on again until he had been removed from it. 'Dark' trials were conducted in complete darkness: all sources of visible light in the laboratory were eliminated and the experimenter used infra-red night vision goggles to conduct the experiments. In a sub-set of experiments, 'light-dark' trials were conducted: in these trials, mice were placed in the arena with the lights turned on, after 10 mins the lights were turned off, and recording continued for a further 20 mins (see Figure S1O-Y).

Rate maps and gridness score. Spike sorting was performed offline using an automated clustering algorithm (KlustaKwik [S1]) followed by a manual review and editing step using an interactive graphical tool (waveform, http://d1manson.github.io/waveform/). After spike sorting, firing rate maps were constructed by binning animals' positions into 1.5 x 1.5cm bins, assigning spikes to each bin, smoothing both position maps and spike maps separately using a 5x5 boxcar filter, and finally dividing the smoothed spike maps by the smoothed position maps. Then spatial autocorrelograms were calculated from the rate maps according to [S2]:

$$r(\tau_x,\tau_y) = \frac{n\sum\lambda(x,y)\lambda(x-\tau_x,\ y-\tau_y) - \sum\lambda(x,y)\sum\lambda(x-\tau_x,\ y-\tau_y)}{\sqrt{n\sum\lambda(x,y)^2 - (\sum\lambda(x,y))^2}.\sqrt{n\sum\lambda(x-\tau_x,\ y-\tau_y)^2 - (\sum\lambda(x-\tau_x,\ y-\tau_y))^2}}$$

Where $r(\tau_x,\tau_y)$is the autocorrelation between bins with spatial offset of τ_x and τ_y. $\lambda(x,y)$ is firing rate in bin (x,y) and n is the number of bins. Six closest peaks of the autocorrelogram were defined by finding six local maxima, closest to (but excluding) the central peak. Gridness was calculated by defining a mask on the spatial autocorrelogram centred on the central peak, but excluding the peak itself (from the centre to the half-height), bounded by a circle with the distance from the central peak to the furthest peak of the six closest peaks. The masked area was rotated in 30° increments up to 150°, and for each rotation the Pearson product moment correlation coefficient was calculated against the un-rotated mask. Gridness was then expressed as the lowest correlation obtained for rotations of 60° and 120° minus the highest correlation obtained at 30°, 90° or 150°.

Grid cell inclusion criterion. Cells were classified as grid cells if their gridness score in 'light' trial exceeded the 95th percentile of a distribution of 1000 gridness scores derived by spatially shuffling the spike data for that cell. To spatially shuffle data for each cell, a fixed time offset was added to all spike times for that cell, and those spike times which then occurred after the end of the trial were 'wrapped' onto the beginning of the trial, by subtracting from their times the trial duration. Position data was unchanged. In this way, the temporal dynamics of the spike train were preserved, but the relation of the spikes to position was uncoupled. Following this procedure, rate maps were constructed and gridness scores calculated as described above. To generate a population of 1000 spatially shuffled gridness scores for each cell, the above procedure was repeated using a set of 1000 offsets, evenly spaced ranging from 1s to trial duration minus 1s.

Measures of stability and spatial tuning. *Intra-trial stability* was measured by correlating the firing rates of spatially corresponding bins from the first and the second halves of a trial, using only those bins in which firing rate > 0 Hz in both halves of the trial.

Spatial information was calculated following [S3]. The estimate of the mutual information $I(R|X)$ between firing rate R and location X is:

$$I(R|X) = \sum_i p(\vec{x_i}) f(\vec{x_i}) \log_2(\frac{f(\vec{x_i})}{F})$$

where $p(\vec{x_i})$ is the probability for the animal being at location (or facing direction) $\vec{x_i}$, $f(\vec{x_i})$ is the firing rate observed at $\vec{x_i}$, and F is the overall firing rate of the cell. $I(R|X)$ was then divided by the overall mean firing rate of the cell in the trial, giving an estimate in bits/spike.

Rayleigh vector (head direction cells). Directional firing rate maps were constructed analogously for those of 2-dimensions, using 6° bins and a 30° wide boxcar smoothing filter. The directional tuning of the cell was measured using the length of the mean resultant vector (Rayleigh Vector; RV) of the bins of directional firing rate map. A cell was defined as a head direction (HD) cell if it displayed an RV ≥ 0.5 in the 'light' trial. HD cells were classified as 'stable dark' HD cells (SD-HD cells) if their RV in the dark trial was reduced by an amount less than or equal to the overall standard deviation of the RV scores in the light (≤0.12).

Speed modulation of theta frequency and firing rate. *Theta frequency.* After band-pass filtering (between 5 and 11Hz), the Hilbert transform was used to define an instantaneous phase for each data point in the filtered LFP signal, and instantaneous phases were 'unwrapped', such that there were no sudden jumps between 0 and 2 π. Instantaneous frequency for LFP sample N was then defined as (phase(N)-phase(N-1))/(2π*sample_rate). The theta frequency for each position sample (20ms duration) was then calculated as the mean of the instantaneous frequencies from the corresponding time points. Position samples were then sorted on the basis of running speed (5cm/s bins running between 5cm/s and 35cm/s), and the overall mean frequency for each speed bin calculated.

Firing rate. First, speed-modulated grid cells were classified from the general population of grid cells following [S4]. Briefly, the degree of speed modulation for each grid cell was characterised by first defining the instantaneous firing rate of the cell as the number of spikes occurring in each position bin divided by the sampling rate. This estimate of instantaneous rate was then smoothed using a 400ms long boxcar filter (matching that applied to position, see above). Finally, a linear correlation was computed between the running speeds and firing rates across all position samples in a trial, and the resulting r-value was taken to characterise the degree of speed modulation for the cell. To be defined as speed-modulated, the r-value for a grid cell had to exceed the 99th percentile of a population of r-values derived by spatially shuffling the spike train. Spatial shuffling was performed in the same way as for calculation of gridness, see 'Grid cell inclusion criterion, above. After speed-modulated grid cells were defined (those grid cells speed-modulated in either the light, or the dark, or both), the nature of speed modulation in the light or the dark was described by sorting position samples on the basis of running

speed, and calculating the mean firing rate, for each cell and for each speed bin, based on the smoothed estimates of instantaneous firing described above.

Coincidence ratio and temporal cross-correlograms. For each pair of simultaneously recorded grid cells, A and B, we identified the sets of spikes from cell B falling within ±2s of each spike in cell A. Then, by subtracting the time of the relevant A spike, the collection of times from cell B were expressed as deltas. The temporal cross-correlograms shown in Figure 3 display this list of deltas as a histogram with 50ms bins, with the counts normalised by the total number of spikes from cell A and by the bin width. Thus, the y-axis values give the mean, over all spikes from cell A, of the rate of cell B at the given temporal offset from a spike in cell A.

To compute the "coincidence-ratio" from the temporal autocorrelogram, we took the mean of the section with $-0.5s < \Delta t < +0.5s$, and divided by the mean of the two sections with $-1.5s < \Delta t < -1s$ and $1s < \Delta t < 1.5s$. The preservation of coincident/non-coincident firing relationships in the dark was then tested using linear regression between the light and dark ratios.

Time-windowed spatial displacement firing rate maps. A time-windowed spatial displacement firing rate map was used to assess short-term spatial structure in grid cell firing in the dark. For each spike, the 2-dimensional displacements of other spikes fired, and positions occupied, within T seconds were counted, and the histograms of displacement were formed for all spikes (bin size 2.4x2.4cm) and smoothed (3x3 boxcar). The histograms for spikes and positions were divided to estimate the probability of spike pairs per second as a function of their displacement. Displacements represented by fewer than 2s of occupancy were not shown. Gridness of time-windowed auto-correlograms was assessed using the gridness measure described above, with the exception that, the six closest peaks were not defined, rather, the gridness mask derived from the whole trial auto-correlogram was used instead. This was found to increase the gridness scores of time-windowed auto-correlograms, as the six closest peaks were not clearly defined in all cases. 1-dimensional distance time-windowed maps were constructed exactly as for the 2D maps described above, but x and y values were collapsed to Pythagorean distance before constructing a 1D rate map, which was smoothed with a 3 bin long boxcar kernel. Exactly as for 2D maps, the firing rate of the real data was re-expressed as standard deviations above the mean firing rate of a set of 100 time-windowed firing rate maps derived from spatially-shuffled data (spatial shuffling performed exactly as described above under "Grid cell inclusion criterion"). This normalisation was performed separately for each spatial bin in the rate map. Lastly, the distance scale of time-windowed distance maps was normalised such that a distance of 1 was equal to the mean grid cell wavelength in the light, for that ensemble of co-recorded cells (as all ensembles consisted of one grid module only, averaging across the ensemble provided a more reliable estimate of grid wavelength). For any given cell, the same normalisation factor was applied to light and dark time-windowed maps.

References

1. Kadir, S. N., Goodman, D. F. M., and Harris, K. D. (2014). High-dimensional cluster analysis with the masked EM algorithm. Neural Comput. *26*, 2379–94.

2. Sargolini, F., Fyhn, M., Hafting, T., McNaughton, B. L., Witter, M. P., Moser, M. B., and Moser, E. I. (2006). Conjunctive representation of position, direction, and velocity in entorhinal cortex. Science (80-.). *312*, 758–762.

3. Skaggs, W. E., McNaughton, B. L., Gothard, K. M., and Markus, E. J. (1993). An information-theoretic approach to deciphering the hippocampal code. Adv Neural Inf Process Syst *5*, 1030–1037.

4. Kropff, E., Carmichael, J. E., Moser, M.-B., and Moser, E. I. (2015). Speed cells in the medial entorhinal cortex. Nature *523*, 419–24.
