# Supplementary material for: Absence of Visual Input Results in the Disruption of Grid Cell Firing in the Mouse
Source: Curr Biol. 2016 Sep 12;26(17):2335–42. doi: 10.1016/j.cub.2016.06.043 (PMC5026695; doi:10.1016/j.cub.2016.06.043)
Supplement: Document S2. Article plus Supplemental Information [file mmc2.pdf]

Current Biology

Report

Absence of Visual Input Results in the Disruption of Grid Cell Firing in the Mouse

Highlights

- Grid cell firing patterns are disrupted in darkness in the mouse
- Grid cells are disrupted even when head direction cell signaling is preserved
- Absence of visual input alters movement velocity modulation of theta frequency
- Temporal firing relationships between grid cell pairs are preserved in the dark

Authors

Guifen Chen, Daniel Manson, Francesca Cacucci, Thomas Joseph Wills

Correspondence

guifen.chen@ucl.ac.uk (G.C.), f.cacucci@ucl.ac.uk (F.C.), t.wills@ucl.ac.uk (T.J.W.)

In Brief

Chen et al. show in the mouse that lack of visual input results in severe disruption of grid cell firing, and that this is not caused by instability in the head direction signal but may be due to changed velocity signaling. Temporal firing relationships between grid cells are preserved, even when their spatial firing patterns are disrupted.

Chen et al., 2016, Current Biology *26*, 2335–2342
September 12, 2016 © 2016 The Author(s). Published by Elsevier Ltd.
http://dx.doi.org/10.1016/j.cub.2016.06.043

CellPress

Current Biology
Report

Absence of Visual Input Results in the Disruption of Grid Cell Firing in the Mouse

Guifen Chen,[1,*] Daniel Manson,[2,3] Francesca Cacucci,[1,*] and Thomas Joseph Wills[2,*]
[1]Department of Neuroscience, Physiology, and Pharmacology, UCL, Gower Street, London WC1E 6BT, UK
[2]Department of Cell and Developmental Biology, UCL, Gower Street, London WC1E 6BT, UK
[3]Centre for Mathematics and Physics in the Life Sciences and Experimental Biology, UCL, Gower Place, London WC1E 6BT, UK
*Correspondence: guifen.chen@ucl.ac.uk (G.C.), f.cacucci@ucl.ac.uk (F.C.), t.wills@ucl.ac.uk (T.J.W.)
http://dx.doi.org/10.1016/j.cub.2016.06.043

SUMMARY

Grid cells are spatially modulated neurons within the medial entorhinal cortex whose firing fields are arranged at the vertices of tessellating equilateral triangles [1]. The exquisite periodicity of their firing has led to the suggestion that they represent a path integration signal, tracking the organism's position by integrating speed and direction of movement [2–10]. External sensory inputs are required to reset any errors that the path integrator would inevitably accumulate. Here we probe the nature of the external sensory inputs required to sustain grid firing, by recording grid cells as mice explore familiar environments in complete darkness. The absence of visual cues results in a significant disruption of grid cell firing patterns, even when the quality of the directional information provided by head direction cells is largely preserved. Darkness alters the expression of velocity signaling within the entorhinal cortex, with changes evident in grid cell firing rate and the local field potential theta frequency. Short-term (<1.5 s) spike timing relationships between grid cell pairs are preserved in the dark, indicating that network patterns of excitatory and inhibitory coupling between grid cells exist independently of visual input and of spatially periodic firing. However, we find no evidence of preserved hexagonal symmetry in the spatial firing of single grid cells at comparable short timescales. Taken together, these results demonstrate that visual input is required to sustain grid cell periodicity and stability in mice and suggest that grid cells in mice cannot perform accurate path integration in the absence of reliable visual cues.

RESULTS

In order to determine the importance of visual cues in supporting grid cell firing, we recorded 277 grid cells from the medial entorhinal cortex after mice were introduced into a familiar environment in total darkness. In the absence of visual cues, the characteristic periodicity of grid cells was disrupted (dark condition; Figure 1A) and gridness scores were considerably reduced compared to the baseline light trials (Figures 1B and S1A; 2 × 2 ANOVA, main effect of light condition, N = 277, $F_{1,275} = 954.22$, $p < 0.001$). Spatial information and intra-trial stability also decreased significantly in darkness (Figures 1C, 1D, S1B, and S1C; 2 × 2 ANOVA, main effect of light condition: spatial information, $F_{1,275} = 372.94$, $p < 0.001$; intra-trial stability, $F_{1,275} = 800.75$, $p < 0.001$). Repeated exposures to the familiar environment in the dark did not rescue the deficit, with the disruption in grid firing patterns persisting even after four or more exposures to the familiar environment in the dark (Figures 1B and S1A; 2 × 2 ANOVA, interaction light × experience, $F_{1,275} = 0.001$, $p = 0.991$). Similarly, spatial information scores in darkness did not improve upon repeated exposures to the dark condition, despite increases in baseline spatial information scores in the light (Figures 1C and S1B; 2 × 2 ANOVA experience, $F_{1,275} = 8.8$, $p = 0.03$; light × experience, $F_{1,275} = 12.8$, $p < 0.001$; simple main effects [SME] $\text{experience}_{(light)}$, $p = 0.001$; SME $\text{experience}_{(dark)}$, $p = 0.167$); while intra-trial stability increased slightly after repeated exposures, in both light and dark (Figures 1D and S1C; 2 × 2 ANOVA experience, $F_{1,275} = 47.7$, $p < 0.001$; light × experience, $F_{1,275} = 3.49$, $p = 0.063$). These results are robust to controlling for the resampling of neurons across days (see Figures S1D–S1F and Supplemental Experimental Procedures for details), and the position and speed sampling did not differ between light and dark, excluding these as potential sources of bias (see Figures S1L–S1N). A subset of grid cells retained above-chance gridness in the dark (defined as a gridness above the 95% confidence-level threshold used to define neurons as grid cells; see Figures S1A and S1G–S1K and Experimental Procedures), but even these grid cells nevertheless showed significant reductions in gridness, spatial information, and spatial stability in darkness (Figure S1J).

To establish whether eliminating visual input disrupts grid firing even when a continuous stream of self-motion information is available, we introduced mice to the familiar environment with the lights on and turned the lights off 10 min after the start of the trial (light-dark condition; Figures S1O–S1Y). Overall, there was no significant improvement over the dark condition: gridness was still significantly lower during the dark phase of the trial compared to the light phase (Figure S1Q; 2 × 2 ANOVA, main effect of light condition, $F_{1,346} = 835.33$, $p < 0.001$). However, after several exposures to the light-dark condition ($n \geq 4$ exposures), gridness values slightly improved, with some regularity appearing in the firing-rate maps (light × experience, $F_{1, 346} = 37.3$, $p < 0.001$; SME $\text{experience}_{(dark)}$, $p = 0.006$; see Figures S1Q–S1S for further quantification).

This is an open access article under the CC BY license (http://creativecommons.org/licenses/by/4.0/).

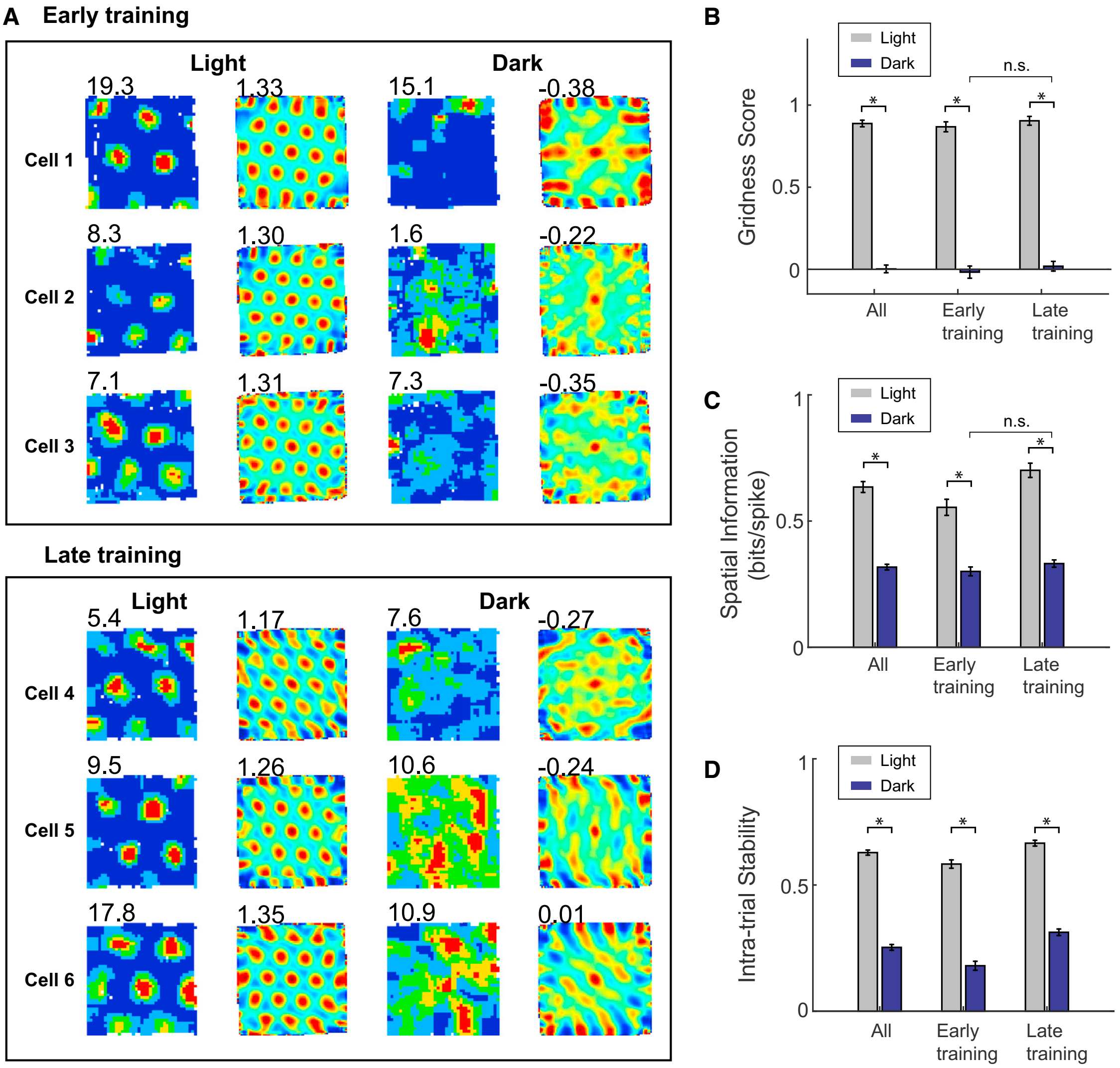

Figure 1. Disruption of Grid Patterns in Complete Darkness

(A) Rate maps (left) and spatial auto-correlograms (right) for six representative grid cells simultaneously recorded in a 60 cm square The top three cells (early training) were recorded during the second exposure to the environment in the dark; the bottom three cells (late training) were recorded during the fifth exposure. The leftmost two columns show data from baseline trials in light; the rightmost two columns show trials in darkness. Numbers at the upper left of firing-rate maps are peak firing rate (Hz); those at the upper left of the auto-correlograms are gridness values.

(B–D) Comparisons of firing properties of grid cells between light trials (gray) and dark trials (blue): gridness (B), spatial information (C), and intra-trial stability (D). Each bar chart shows the mean values (±SEM) for all recorded grid cells (left group of bars), those recorded during days 1–3 of exposure to darkness (middle group), and those recorded during days 4–9 of exposure to darkness (right group). *$p < 0.001$ level; n.s., $p > 0.05$.

See also Figure S1.

A stable directional heading signal from the head direction cell circuit is necessary for generating and maintaining grid cells' regular firing [11], and there is evidence that darkness can induce head direction instability in mice [12]. In order to test whether grid cell disruption in the dark is caused by directional instability, we obtained simultaneous recordings of head direction and grid cells (number of HD cells = 34, number of ensembles with simultaneously recorded HD and grid cells = 17; see Figure 2A for examples). As a measure of head direction (HD) cell stability within a trial, we computed the Rayleigh vector (RV) score, with cells considered to exhibit stable directional tuning in the dark if the $\Delta RV_{light-dark}$ fell within one standard deviation of the RV scores for the whole HD cell population during the light trial ($\Delta RV_{light-dark} \leq 0.12$). Of the 34 HD cells recorded, 22 cells (77%) were classified as stable-HD (with 12/17 [70%] grid cell/HD cell ensembles containing at least one stable-HD; see Figures S2A and S2C). These stable-HDs continued to show strong directional tuning in the absence of visual inputs (Figure 2B; 2 × 2 ANOVA light × HD stability, RV: main effect of light, $F_{1,32} = 93.0$, $p < 0.001$; HD stability $F_{1,32} = 7.6$, $p = 0.009$; light ×HD stability, $F_{1,32} = 48.4$, $p < 0.001$; SME HD stability$_{(light)}$ $p = 0.76$; SME HD stability$_{(dark)}$ $p < 0.001$). In some cases, stable-HDs showed the same preferred firing direction in darkness as those in the light session (with 4/12 ensembles displaying an average preferred

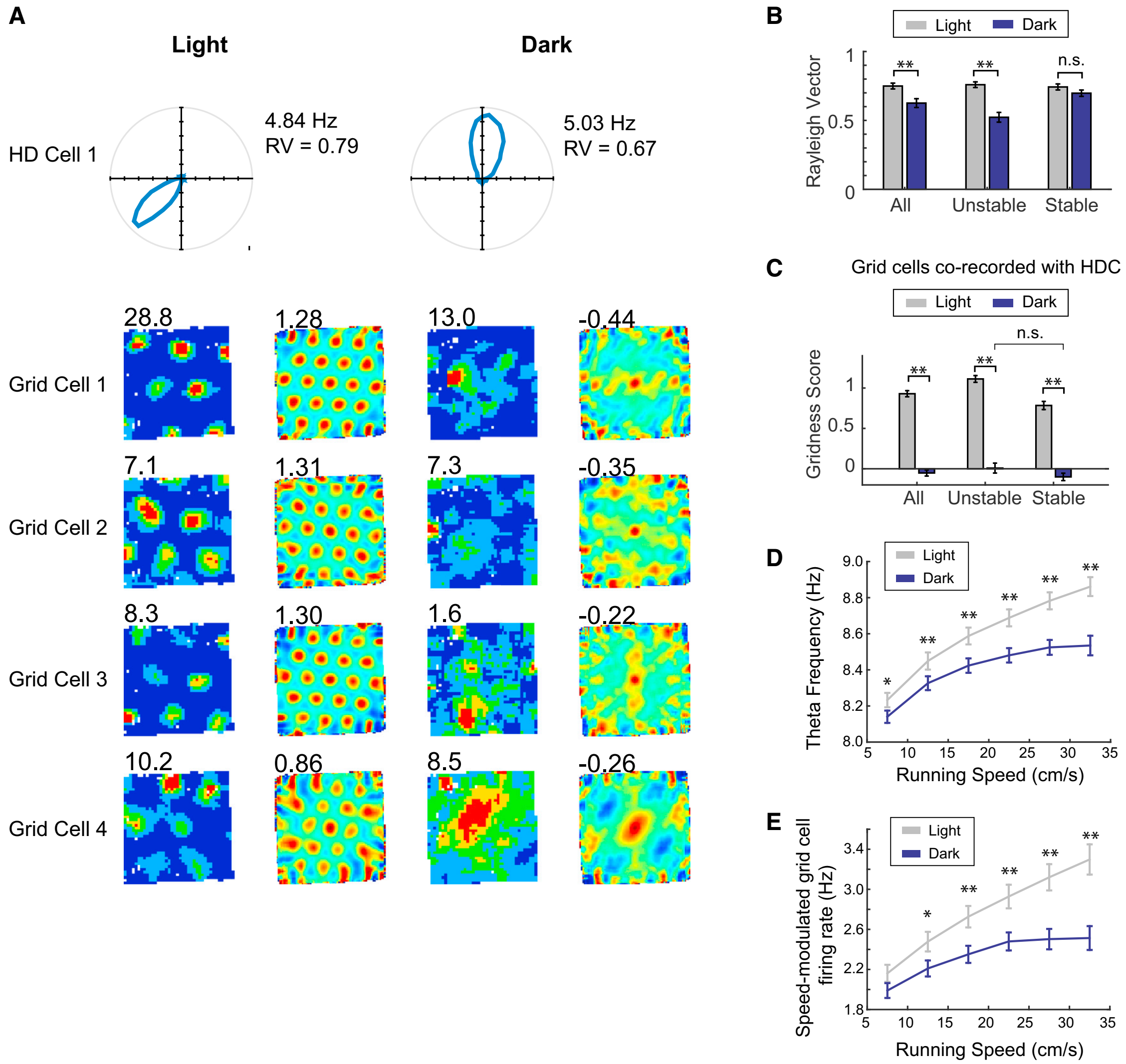

Figure 2. Disruption of Grid Patterns in Darkness Is Accompanied by Stable Directional Input but Altered Velocity Signaling

(A) Polar plots for a representative head direction cell showing directional tuning in darkness and firing-rate maps and auto-correlograms for simultaneously recorded grid cells in light trials (left columns) and dark trials (right columns). Numbers at the upper right of polar plots are peak firing rate (Hz) and Rayleigh vector (RV), those at the upper left of firing-rate maps are peak firing rate (Hz), and those at the upper left of auto-correlograms are gridness values.

(B) Mean (±SEM) RV scores of all recorded HD cells (n = 34), HD cells that are unstable in the dark (n = 12), and those that are stable in the dark (n = 22).

(C) Mean (±SEM) gridness scores of grid cells co-recorded with unstable HD cells and stable HD cells in light (gray) and dark (blue).

(D) Relationship between running speed and instantaneous theta frequency of entorhinal cortex local field potential in light (gray) and dark (blue). Lines show the mean (±SEM) instantaneous frequency in each running speed bin (5 cm/s to 35 cm/s in 5 cm/s bins).

(E) Relationship between running speed and firing rates of speed-modulated grid cells in light (gray) and dark (blue). Lines show the mean (±SEM) firing rate of all speed-modulated grid cells in each running speed bin.

**p < 0.001; *p < 0.05; n.s., p > 0.05; post hoc differences between light and dark for each speed bin. See also Figure S2.

direction shift < 25°; see Figures S2B and S2C; note that in all cases, pairs of simultaneously recorded HD cells kept consistent preferred direction offsets; see Figure S2B). Grid cells simultaneously recorded with at least one stable-HD showed the same degree of disruption in the dark (with a trend toward greater disruption) as those recorded with unstable-HDs (Figure 2C; n = 88 grid cells; 2 × 2 ANOVA light × HD stability, gridness: main effect of light, $F_{1,86} = 321$, $p < 0.001$; HD stability $F_{1,86} = 21.0$, $p < 0.001$; light × HD stability, $F_{1,86} = 3.70$, $p = 0.043$; SME stability$_{(dark)}$ $p = 0.076$). These results are robust to controlling for the resampling of neurons across days (see Figures S2D and S2E). These data show that, in the mouse, absence of visual input results in a larger degree of disruption to regular grid cell firing patterns than to HD signaling.

We also co-recorded with grid cells a small number of putative boundary-responsive (BR) cells [13, 14] (n = 8). Interestingly, we observed a qualitatively similar degree of disruption of BR cells' firing to that of grid cells, with both spatial information and within-trial stability scores of BR cells dropping in the dark (see Figures S2F and S2G).

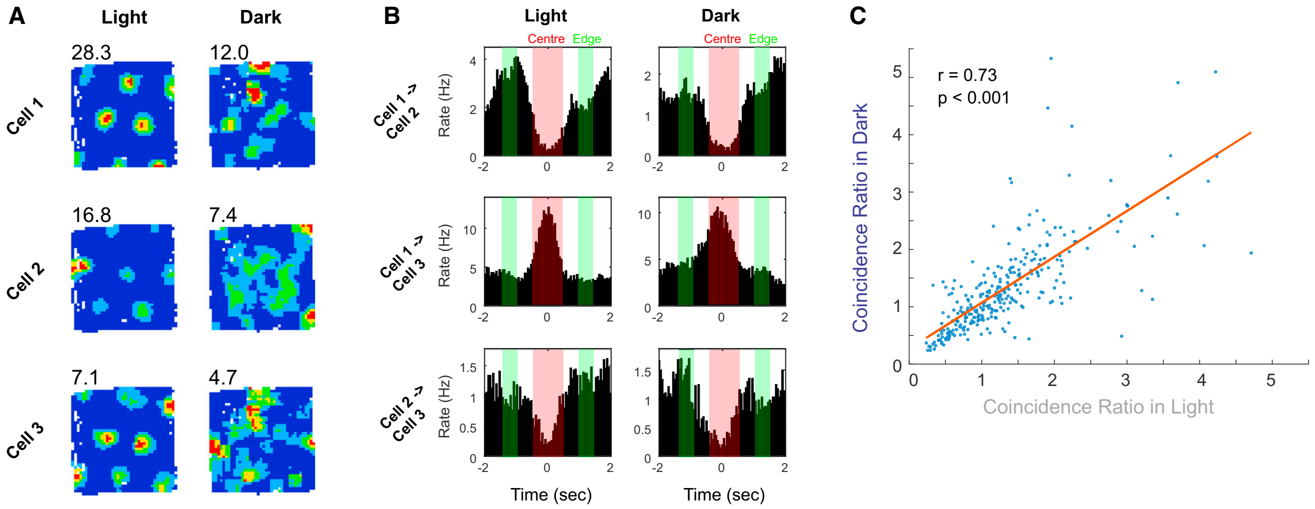

Figure 3. Preserved Temporal Cross-correlations between Grid Cell Pairs in Darkness

(A) Rate maps of three representative grid cells in light (left column) and in darkness (right column).

(B) Example temporal cross-correlograms between each pairing of the three cells shown (A) in light (left column) and in darkness (right column). The time windows used to calculate the coincidence ratio of the cross-correlogram are shown as transparent colored boxes (center in red; edge in green).

(C) The coincidence ratios of all simultaneously recorded grid cell pairs in darkness were significantly correlated with those in light ($r = 0.73$, $p < 0.001$).

See also Figure S3.

As we discounted heading instability as the sole source of grid cell disruption, we sought to identify other potential sources of positional error. The two main classes of grid cell model (continuous attractor and oscillatory interference) both include a velocity signal that allows the static grid cell representation to be generated and updated on the basis of the organism's displacement [2–10]. Velocity information may be carried, in the hippocampus, by the frequency of theta, the principal oscillation observed in the local field potential during movement (with theta frequency increasing monotonically with running speed [15, 16]). We found a reduction in the increase of theta frequency with running speed in the dark (Figure 2D; 2 × 6 ANOVA light × speed; main effect of speed, $F_{5,216} = 22.4$, $p < 0.001$; light, $F_{1,216} = 334$, $p < 0.001$; light × speed $F_{5,216} = 8.13$, $p < 0.001$). Theta frequency in light was significantly greater than frequency in the dark at all speed values (SME light-dark $p < 0.05$ for all). However, although reduced, speed modulation of frequency was still present in darkness, as shown by significant post hoc differences between frequency in the speed bins 32.5 cm/s and 17.5 cm/s (SME_{dark}; $p = 0.042$), or between 32.5 cm/s and 12.5 cm/s or slower ($p < 0.001$). Another potential source of velocity signal to the grid cell network is the speed modulation of firing rate of entorhinal cortex neurons, both "speed cells" [17] and/or speed-modulated grid cells [18, 19]. 160 grid cells (58%) were classified as speed-modulated (see Experimental Procedures). The speed modulation of these cells' firing was altered in the dark: the degree of firing-rate increase with speed was reduced, and firing rate in the dark was significantly less than that in the light from speeds of greater than 10 cm/s onward (Figure 2E; 2 × 6 ANOVA speed × light: main effect of speed, $F_{5,954} = 11.8$, $p < 0.001$; light, $F_{1,954} = 100.0$, $p < 0.001$; light × speed $F_{5,903} = 4.32$, $p = 0.001$; SME light significant for all speed bins ≥ 12.5 cm/s, $p < 0.05$). As for frequency, significant differences in rate were present between speed bins 32.5 cm/s and 12.5 cm/s (SME dark; $p = 0.021$) even in darkness, showing that speed modulation is reduced, but not eliminated, in the dark. Taken together, these results indicate that the animal's estimation of speed is likely altered by the absence of visual input. It is possible that, similarly to what is found under passive transport conditions [20], the combination of relatively accurate computation of angular displacement (conveyed by the only mildly altered HD signaling) and more significantly altered computation of linear displacement (due to the reduction of theta frequency and grid cell firing dependency on running speed) may be the cause of grid cell disruption in the dark in the mouse.

Several models of grid cell firing posit that grid cells are arranged as a low-dimensional continuous attractor [6–8, 10]. This network structure implies that excitatory/inhibitory relationships between grid cell pairs are fixed and should be preserved even if the network becomes decoupled from external sensory inputs [21, 22]. To assess whether grid cells showed coincident firing (indicative of excitatory coupling) or offset firing (indicative of inhibitory coupling), we constructed temporal cross-correlograms of grid cell pairs' spike trains and calculated the "coincidence ratio" between the mean rate within the central (±0.5 s) and offset (±1–1.5 s) portions of the correlogram (see Figures 3A and 3B for examples; central areas are marked in red and offset areas in green). Coincidence ratios in the light and dark are highly correlated across all cell pairs (Figure 3C; $r = 0.73$, $p < 0.001$), and this strong relationship holds true also when data are analyzed separately for each simultaneously recorded grid cell ensemble (see Figure S3). Co-recorded grid cell pairs therefore show preserved temporal firing relationships even when, in the dark, grid cell representations have lost their spatial stability across the whole trial length.

The temporal cross-correlogram analyses show that temporal grid cell coupling is preserved at short timescales (on the order of ± 1.5 s). We therefore sought to probe whether the spatial

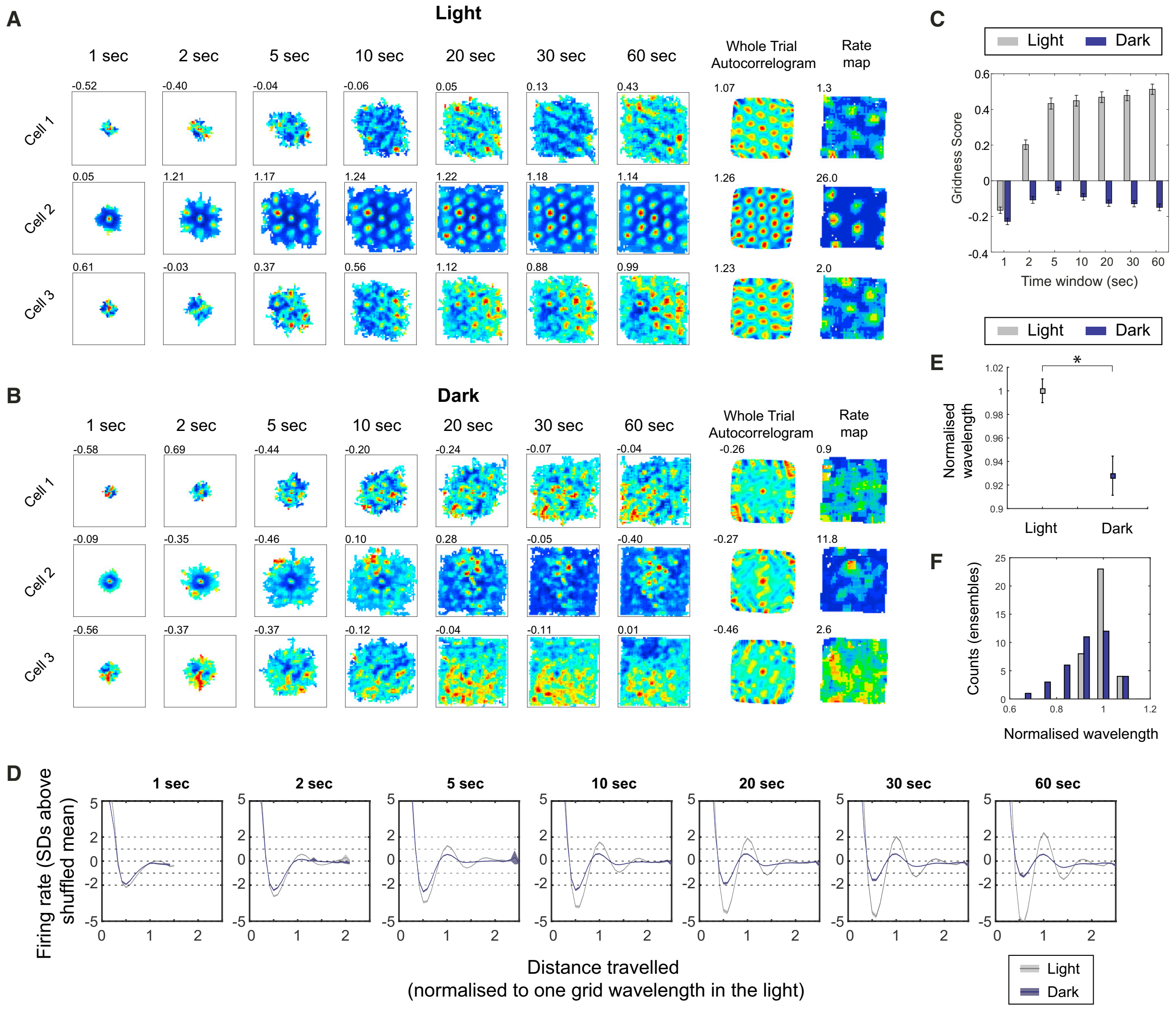

Figure 4. Loss of Hexagonal Symmetry and Compressed Displacement Scale in Grid Cell Firing at Short Timescales in Total Darkness

(A) 2D time-windowed spatial displacement firing-rate maps (1, 2, 5, 10, 20, 30, and 60 s windows; first seven columns) for three representative simultaneously recorded grid cells in light, and respective whole-trial spatial auto-correlograms (eighth column) and firing-rate maps (ninth column). For complete ensemble, see Figures S4A and S4B.

(B) Same set of cells as in (A), recorded in darkness.

(C) Mean (±SEM) gridness scores calculated from the time-windowed maps for time windows of increasing duration, in both light (gray) and dark (blue).

(D) 1D time-windowed spatial displacement firing-rate maps (x and y dimensions collapsed to Pythagorean displacement; 1, 2, 5, 10, 20, 30, and 60 s windows) averaged across all grid cells for light (gray) and dark (blue) conditions. The x axis for each plot shows displacement distance, normalized to the grid wavelength in the light for each cell. The y axes show firing rate expressed as SD above the mean of a spatially shuffled control (see Supplemental Experimental Procedures). Shaded areas around the lines show the SEM at each spatial bin.

(E) Mean (±SEM) of the wavelengths of time-windowed distance maps (means for each simultaneously recorded ensemble) in both light and dark. Wavelength was defined as the distance to the first peak of the distance map after zero. $^{*}p \leq 0.01$. Data here are based on a 20 s time window.

(F) Histogram showing the raw data contributing to the means in (E), i.e., the distributions of ensemble mean wavelengths in light (gray) and dark (blue).

See also Figure S4.

structure of each grid cell's firing is preserved at comparable timescales, by computing time-windowed spatial displacement firing-rate maps of individual grid cells for both light and dark trials (similarly to [23]). We found no evidence of preserved hexagonal symmetry at timescales ranging from 1 to 60 s (Figures 4A–4C). To test whether grid cells continued to code for distance traveled at short timescales in the dark, we re-computed the time-windowed spatial displacement firing-rate maps, collapsing the x and y dimensions into a single "displacement distance" metric (1D displacement firing-rate maps; see Figure 4D and Supplemental Experimental Procedures for details). We found that a degraded signal for distance traveled

was preserved at short timescales in the dark (Figure 4D). Notably, the scale of the distance code (as measured by the distance from the origin to the first peak of the 1D displacement map) was compressed, such that overall the distance between firing peaks was approximately 7% shorter in the dark, at a time window of 20 s (see Figures 4E and 4F; paired t test: $t_{(36)} = 3.76$, $p = 0.001$). The displacement scale was also significantly smaller in the dark at time windows of 10 s (96.4% of normalized wavelength, $t_{(36)} = 2.25$, $p = 0.031$) and 30 s (94.2% of normalized wavelength, $t_{(36)} = 2.21$, $p = 0.033$), but not at 1 s, 2 s, 5 s, or 60 s (data not shown).

DISCUSSION

Grid cells are widely thought to integrate self-motion information and to provide the rest of the brain with a path integrative input that can be used to calculate one's location in space even when external sensory input is lacking or too noisy [24, 25]. This view is supported by the finding in the rat that grid cell patterns are preserved when grid cells are recorded in darkness [1, 26]. Surprisingly, we show here that when grid cells are recorded in the mouse in the dark, under very similar conditions to the studies reported above, grid cell firing is severely disrupted (Figure 1). Turning off the lights while the mouse is in the arena (Figures S1O–S1Y) allows a partial rescue of this disruption, but only after several training trials in darkness, indicating that with sufficient training, animals might learn strategies to remain oriented (possibly exploiting olfactory traces left during the initial, illuminated, part of the trial). These data indicate that in mice, grid cells may not always be able to provide an accurate estimate of position solely on the basis of self-motion cues, suggesting that homing abilities in darkness in the mouse (of the kind tested by [27]) might not require stable and periodic grid cell firing. These results are at odds with those obtained in the rat [1, 26] and raise interesting questions as to the source (or sources) of this striking inter-species difference in the resilience of grid signaling to removal of visual cues.

Importantly, we have excluded heading disorientation as the sole and/or critical cause of grid cell disruption. The degree of disruption to the HD signal is not qualitatively comparable to that observed in either grid or border cells. Most HD cells (77%) were stable in the dark. Importantly, even when the HD preferred direction did not change between the light and dark trials, grid cell firing was disrupted and grid symmetry lost in the dark. Such an uncoupling between grid and head direction cells has recently been described in the rat, with passive transportation also selectively affecting grid cell firing patterns while largely sparing head direction firing [20].

The discovery of the grid cell phenomenon and the regularity of grid cells' firing has inspired a large number of modeling efforts [2–10, 28], which all share the central idea that grid cells must receive a velocity signal in order to allow the spatial location to be updated on the basis of the animal's displacement. The source of this velocity signal has been posited to be either the frequency of the theta oscillation or the firing rate of grid cells and speed cells (the three increase linearly with running speed [15–19]). Interestingly, we have demonstrated here that the relationships between running speed and both theta frequency (Figure 2D) and the firing rate of speed-modulated grid cells (Figure 2E) are both altered when mice explore the familiar environment in the dark. We therefore speculate that optic flow might be an important determinant in the computation of running speed, and that vestibular and proprioceptive information are therefore not sufficient in the mouse to provide an accurate estimate of linear speed (and consequently spatial displacement). This is consistent with a modeling study [29] that demonstrated how optic flow can be used to compute a velocity signal capable of sustaining grid cell firing (see also [30]), and with recent experimental evidence demonstrating that loss of theta frequency and/or firing-rate modulation by speed is associated with disruption of grid signaling [20, 31].

In sum, we observed that darkness may produce small decreases in directional signaling specificity, larger decreases in border cell stability and spatial tuning, and altered signaling of velocity. Future modeling efforts may be useful to establish if and/or how each of these phenomena could contribute to the strong disruption of grid cell firing observed in our study.

The loss of spatial periodicity in grid cell firing in the dark allowed us to probe whether a grid cell network that has lost its stable and regular relationship with external inputs still retains preserved inhibitory and/or excitatory relationships between cell pairs, as postulated by continuous attractor models [6–8, 10]. Consistent with these models, we found that the temporal relationship (at timescales of 1.5 s) between simultaneously recorded grid cell pairs is spared by darkness (Figures 3 and S3). Our results therefore confirm the findings by Yoon and colleagues [22] showing that under conditions where each grid cell response is deformed, the coupling of grid cell pairs is still preserved, and generalize them to the extreme case where grid cell firing patterns are substantially degraded and no longer bear any obvious spatial relationship to external sensory inputs. Despite preserved temporal coupling between grid cells in the dark, we found no evidence in support of the view that single grid cell firing patterns display hexagonal symmetry at comparatively short timescales, although we found evidence suggesting that, in the dark, a distance code, although compressed, is still present in grid cell firing (Figure 4).

In conclusion, we have demonstrated here that, in the mouse, grid cell firing is reliant on visual input. Absence of visual input results in extensive disruption of grid cell periodicity, possibly by altering running speed computation, as reflected by the reduced dependency of theta frequency and grid cell firing on running speed in the dark. Temporal relationships between grid cell pairs are preserved in the dark, supporting the view that grid cell networks are supported by continuous attractor dynamics, and a degraded code for distance is also preserved. However, we find no evidence that the attractor is able to generate regular hexagonal spatial firing in darkness, even on the shortest of measurable timescales.

EXPERIMENTAL PROCEDURES

Subjects and Surgery

Six wild-type mice (C57BL/6J) were implanted with custom-made microdrives, loaded with 17 μm platinum-iridium tetrodes, targeted at the mEC. Tetrodes were implanted 3.0 mm lateral to bregma, 0.2 mm anterior to the transverse sinus, 0.8 mm below the brain surface and angled 4° posteriorly. Electrode position was confirmed postmortem by transcardial perfusion with 4% paraformaldehyde in PBS followed by Nissl staining. All work was

performed according to the Animals (Scientific Procedures) Act 1986 and according to Home Office and institutional guidelines.

Behavioral Training

After recovery from surgery, mice were exposed to the same recording arena every day (20 min per day) to screen for grid cell activity. Electrodes were lowered by 62.5 μm per day until grid cells were found. During all screening and recording sessions, mice foraged for sweetened soy milk drops scattered pseudo-randomly throughout the arena. Trials lasted for 20 min. The floor was cleaned with 70% ethanol between exposures. Experimental sessions began after grid cells were found (after 3–17 screening trials). An experimental session comprised a 20 min trial in the familiar environment in the light and a 20 min trial in the same environment with the lights turned off (lights were turned off before the mouse was placed in the environment). The order of "light" and "dark" trials was counterbalanced across mice and across experience. See Supplemental Experimental Procedures for further details.

Rate Maps: Assessing Gridness and Spatially Tuned Firing

Spike sorting was performed offline using an automated clustering algorithm (KlustaKwik [32]) followed by a manual review. Firing-rate maps constructed using 1.5 × 1.5 cm spatial bins and a 5 × 5 boxcar filter. Spatial auto-correlograms and "gridness" scores were calculated from the rate maps similarly to [18] (see Supplemental Experimental Procedures for further details). Cells were classified as grid cells if their gridness score in "light" trials exceeded the 95th percentile of a distribution of 1,000 gridness scores derived by spatially temporally shuffling the spike data for that cell. Shuffling was performed similarly to [33](see Supplemental Experimental Procedures for further details). For calculation of intra-trial stability, spatial information, and Rayleigh vector, see Supplemental Experimental Procedures.

Speed Modulation of Theta Frequency and Grid Cell Firing Rate

Theta Frequency

Local-field potential signals recorded concurrently with grid cells were band-pass filtered between 5 and 11 Hz. The Hilbert transform was used to define an instantaneous frequency for each position sample. Position samples were then sorted according to running speed (5 cm/s bins), and the mean frequency for each speed bin was calculated.

Firing Rate

Speed-modulated grid cells were defined as in [17]. Following this, for those grid cells that were speed-modulated in either light or dark, the mean firing rate at each speed was defined as the number of spikes occurring at a given speed, divided by the total time during the trial spent moving at that speed. See Supplemental Experimental Procedures for further details.

Coincidence Ratio and Temporal Cross-correlograms

To compute the "coincidence ratio" from the temporal cross-correlogram, we took the mean of the section with $-0.5\ s < \Delta t < +0.5\ s$ and divided by the mean of the two sections with $-1.5\ s < \Delta t < -1\ s$ and $1\ s < \Delta t < 1.5\ s$. The preservation of coincident/non-coincident firing relationships in the dark was then tested using linear regression between the light and dark ratios.

Time-Windowed Spatial Displacement Firing-Rate Maps

Time-windowed spatial displacement firing-rate maps were constructed following [23]. 1D displacement maps were constructed using the same time-windowing procedure, but following conversation of displacements in the x and y dimensions to Pythagorean distance, before constructing rate maps. For both 1D and 2D maps, to reduce the noise caused by low levels of position sampling, 100 spatially shuffled time-windowed spatial displacement maps were calculated (created by shifting the spike train by a random amount of at least 20 s with respect to the position data), and the real-time-windowed firing-rate values were re-expressed as the number of standard deviations above the mean of the shuffled population for each spatially corresponding bin. Gridness of time-windowed auto-correlograms was assessed using the gridness measure described above, with the exception that the six closest peaks were not defined; rather, the gridness mask derived from the whole-trial auto-correlogram was used instead.

SUPPLEMENTAL INFORMATION

Supplemental Information includes four figures and Supplemental Experimental Procedures and can be found with this article online at http://dx.doi.org/10.1016/j.cub.2016.06.043.

AUTHOR CONTRIBUTIONS

G.C., F.C., and T.J.W. designed the experiments and analyses. G.C. collected the data. G.C., D.M., F.C., and T.J.W. analyzed the data. G.C., F.C., and T.J.W. contributed to drafting the manuscript.

ACKNOWLEDGMENTS

We acknowledge funding from the ERC (Starting Grant "DEVSPACE" to F.C.) and the Royal Society (fellowship UF100746 and research grant RG110300 to T.J.W.). We thank Neil Burgess and Caswell Barry for helpful discussions.

Received: April 29, 2016
Revised: May 30, 2016
Accepted: June 20, 2016
Published: August 4, 2016

REFERENCES

1. Hafting, T., Fyhn, M., Molden, S., Moser, M.B., and Moser, E.I. (2005). Microstructure of a spatial map in the entorhinal cortex. Nature *436*, 801–806.
2. Burgess, N., Barry, C., and O'Keefe, J. (2007). An oscillatory interference model of grid cell firing. Hippocampus *17*, 801–812.
3. Blair, H.T., Gupta, K., and Zhang, K. (2008). Conversion of a phase- to a rate-coded position signal by a three-stage model of theta cells, grid cells, and place cells. Hippocampus *18*, 1239–1255.
4. Hasselmo, M.E. (2008). Grid cell mechanisms and function: contributions of entorhinal persistent spiking and phase resetting. Hippocampus *18*, 1213–1229.
5. Welday, A.C., Shlifer, I.G., Bloom, M.L., Zhang, K., and Blair, H.T. (2011). Cosine directional tuning of theta cell burst frequencies: evidence for spatial coding by oscillatory interference. J. Neurosci. *31*, 16157–16176.
6. Fuhs, M.C., and Touretzky, D.S. (2006). A spin glass model of path integration in rat medial entorhinal cortex. J. Neurosci. *26*, 4266–4276.
7. McNaughton, B.L., Battaglia, F.P., Jensen, O., Moser, E.I., and Moser, M.B. (2006). Path integration and the neural basis of the 'cognitive map'. Nat. Rev. Neurosci. *7*, 663–678.
8. Burak, Y., and Fiete, I.R. (2009). Accurate path integration in continuous attractor network models of grid cells. PLoS Comput. Biol. *5*, e1000291.
9. Bush, D., and Burgess, N. (2014). A hybrid oscillatory interference/continuous attractor network model of grid cell firing. J. Neurosci. *34*, 5065–5079.
10. Pastoll, H., Solanka, L., van Rossum, M.C.W., and Nolan, M.F. (2013). Feedback inhibition enables θ-nested γ oscillations and grid firing fields. Neuron *77*, 141–154.
11. Winter, S.S., Clark, B.J., and Taube, J.S. (2015). Spatial navigation. Disruption of the head direction cell network impairs the parahippocampal grid cell signal. Science *347*, 870–874.
12. Yoder, R.M., and Taube, J.S. (2009). Head direction cell activity in mice: robust directional signal depends on intact otolith organs. J. Neurosci. *29*, 1061–1076.
13. Solstad, T., Boccara, C.N., Kropff, E., Moser, M.B., and Moser, E.I. (2008). Representation of geometric borders in the entorhinal cortex. Science *322*, 1865–1868.
14. Lever, C., Burton, S., Jeewajee, A., O'Keefe, J., and Burgess, N. (2009). Boundary vector cells in the subiculum of the hippocampal formation. J. Neurosci. *29*, 9771–9777.

15. Sławińska, U., and Kasicki, S. (1998). The frequency of rat's hippocampal theta rhythm is related to the speed of locomotion. Brain Res. *796*, 327–331.

16. Jeewajee, A., Barry, C., O'Keefe, J., and Burgess, N. (2008). Grid cells and theta as oscillatory interference: electrophysiological data from freely moving rats. Hippocampus *18*, 1175–1185.

17. Kropff, E., Carmichael, J.E., Moser, M.-B., and Moser, E.I. (2015). Speed cells in the medial entorhinal cortex. Nature *523*, 419–424.

18. Sargolini, F., Fyhn, M., Hafting, T., McNaughton, B.L., Witter, M.P., Moser, M.B., and Moser, E.I. (2006). Conjunctive representation of position, direction, and velocity in entorhinal cortex. Science *312*, 758–762.

19. Wills, T.J., Barry, C., and Cacucci, F. (2012). The abrupt development of adult-like grid cell firing in the medial entorhinal cortex. Front. Neural Circuits *6*, 21.

20. Winter, S.S., Mehlman, M.L., Clark, B.J., and Taube, J.S. (2015). Passive Transport Disrupts Grid Signals in the Parahippocampal Cortex. Curr. Biol. *25*, 2493–2502.

21. Fyhn, M., Hafting, T., Treves, A., Moser, M.B., and Moser, E.I. (2007). Hippocampal remapping and grid realignment in entorhinal cortex. Nature *446*, 190–194.

22. Yoon, K., Buice, M.A., Barry, C., Hayman, R., Burgess, N., and Fiete, I.R. (2013). Specific evidence of low-dimensional continuous attractor dynamics in grid cells. Nat. Neurosci. *16*, 1077–1084.

23. Bonnevie, T., Dunn, B., Fyhn, M., Hafting, T., Derdikman, D., Kubie, J.L., Roudi, Y., Moser, E.I., and Moser, M.B. (2013). Grid cells require excitatory drive from the hippocampus. Nat. Neurosci. *16*, 309–317.

24. Poucet, B., Sargolini, F., Song, E.Y., Hangya, B., Fox, S., and Muller, R.U. (2013). Independence of landmark and self-motion-guided navigation: a different role for grid cells. Philos. Trans. R. Soc. Lond. B Biol. Sci. *369*, 20130370.

25. Bush, D., Barry, C., and Burgess, N. (2014). What do grid cells contribute to place cell firing? Trends Neurosci. *37*, 136–145.

26. Barry, C., Ginzberg, L.L., O'Keefe, J., and Burgess, N. (2012). Grid cell firing patterns signal environmental novelty by expansion. Proc. Natl. Acad. Sci. USA *109*, 17687–17692.

27. Yoder, R.M., Goebel, E.A., Köppen, J.R., Blankenship, P.A., Blackwell, A.A., and Wallace, D.G. (2015). Otolithic information is required for homing in the mouse. Hippocampus *25*, 890–899.

28. Schmidt-Hieber, C., and Häusser, M. (2013). How to build a grid cell. Philos. Trans. R. Soc. Lond. B Biol. Sci. *369*, 20120520.

29. Raudies, F., Mingolla, E., and Hasselmo, M.E. (2012). Modeling the influence of optic flow on grid cell firing in the absence of other cues1. J. Comput. Neurosci. *33*, 475–493.

30. Raudies, F., Hinman, J.R., and Hasselmo, M.E. (2016). Modelling effects on grid cells of sensory input during self-motion. J. Physiol. Published online April 20, 2016. http://dx.doi.org/10.1113/JP270649.

31. Jacob, P.-Y., Poucet, B., Liberge, M., Save, E., and Sargolini, F. (2014). Vestibular control of entorhinal cortex activity in spatial navigation. Front. Integr. Nuerosci. *8*, 38.

32. Kadir, S.N., Goodman, D.F.M., and Harris, K.D. (2014). High-dimensional cluster analysis with the masked EM algorithm. Neural Comput. *26*, 2379–2394.

33. Wills, T.J., Cacucci, F., Burgess, N., and O'Keefe, J. (2010). Development of the hippocampal cognitive map in preweanling rats. Science *328*, 1573–1576.

Current Biology, Volume *26*

Supplemental Information

Absence of Visual Input Results in the Disruption of Grid Cell Firing in the Mouse

Guifen Chen, Daniel Manson, Francesca Cacucci, and Thomas Joseph Wills

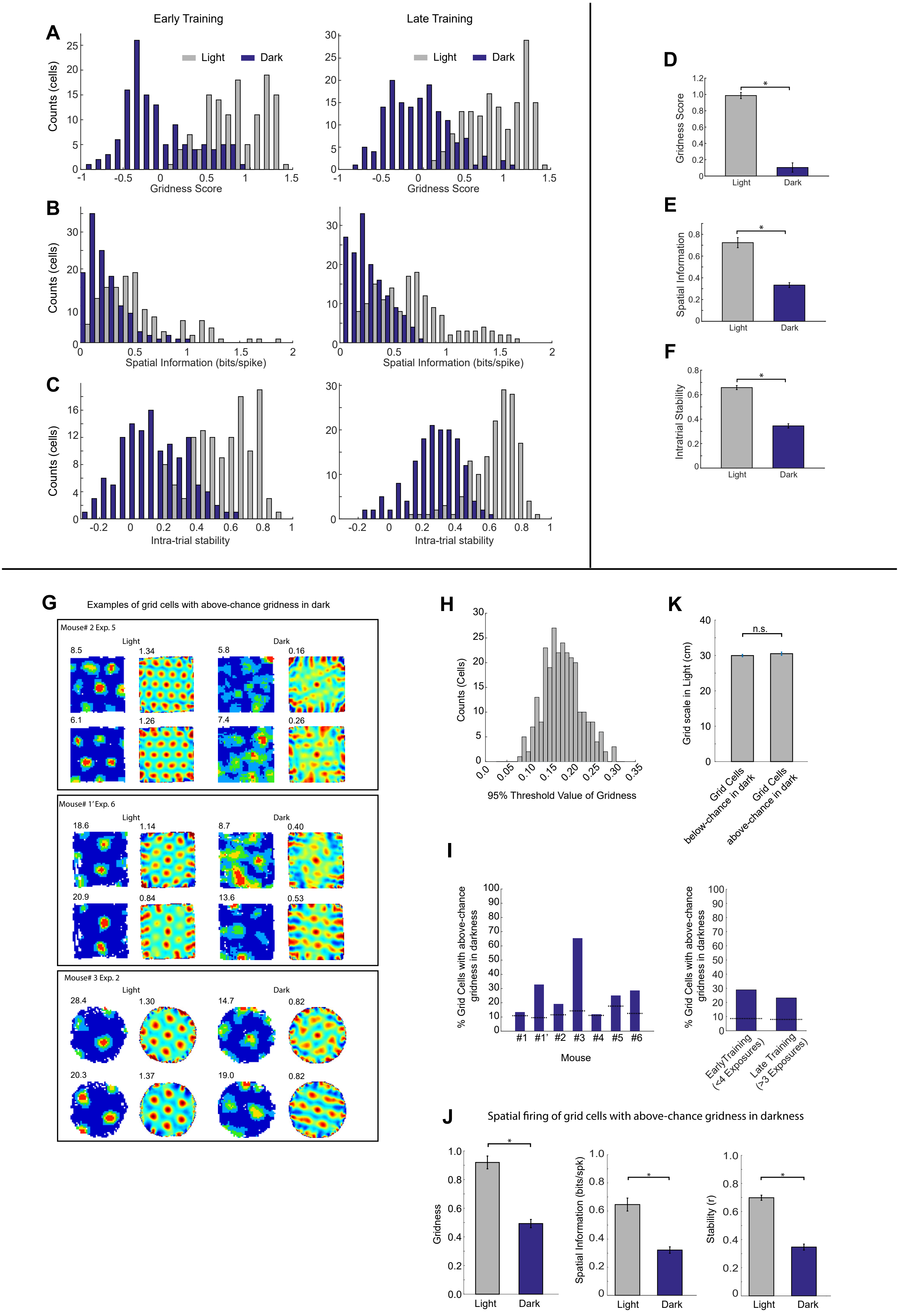
A
Early Training
Late Training
Light
Dark
Counts (cells)
Gridness Score
B
Spatial Information (bits/spike)
C
Intra-trial stability
D
Gridness Score
E
Spatial Information
F
Intratrial Stability
G
Examples of grid cells with above-chance gridness in dark
Mouse# 2 Exp. 5
Mouse# 1' Exp. 6
Mouse# 3 Exp. 2
8.5
1.34
5.8
0.16
6.1
1.26
7.4
0.26
18.6
1.14
8.7
0.40
20.9
0.84
13.6
0.53
28.4
1.30
14.7
0.82
20.3
1.37
19.0
0.82
H
Counts (Cells)
95% Threshold Value of Gridness
K
Grid scale in Light (cm)
n.s.
Grid Cells below-chance in dark
Grid Cells above-chance in dark
I
% Grid Cells with above-chance gridness in darkness
#1
#1'
#2
#3
#4
#5
#6
Mouse
EarlyTraining (<4 Exposures)
Late Training (>3 Exposures)
J
Spatial firing of grid cells with above-chance gridness in darkness
Gridness
Spatial Information (bits/spk)
Stability (r)

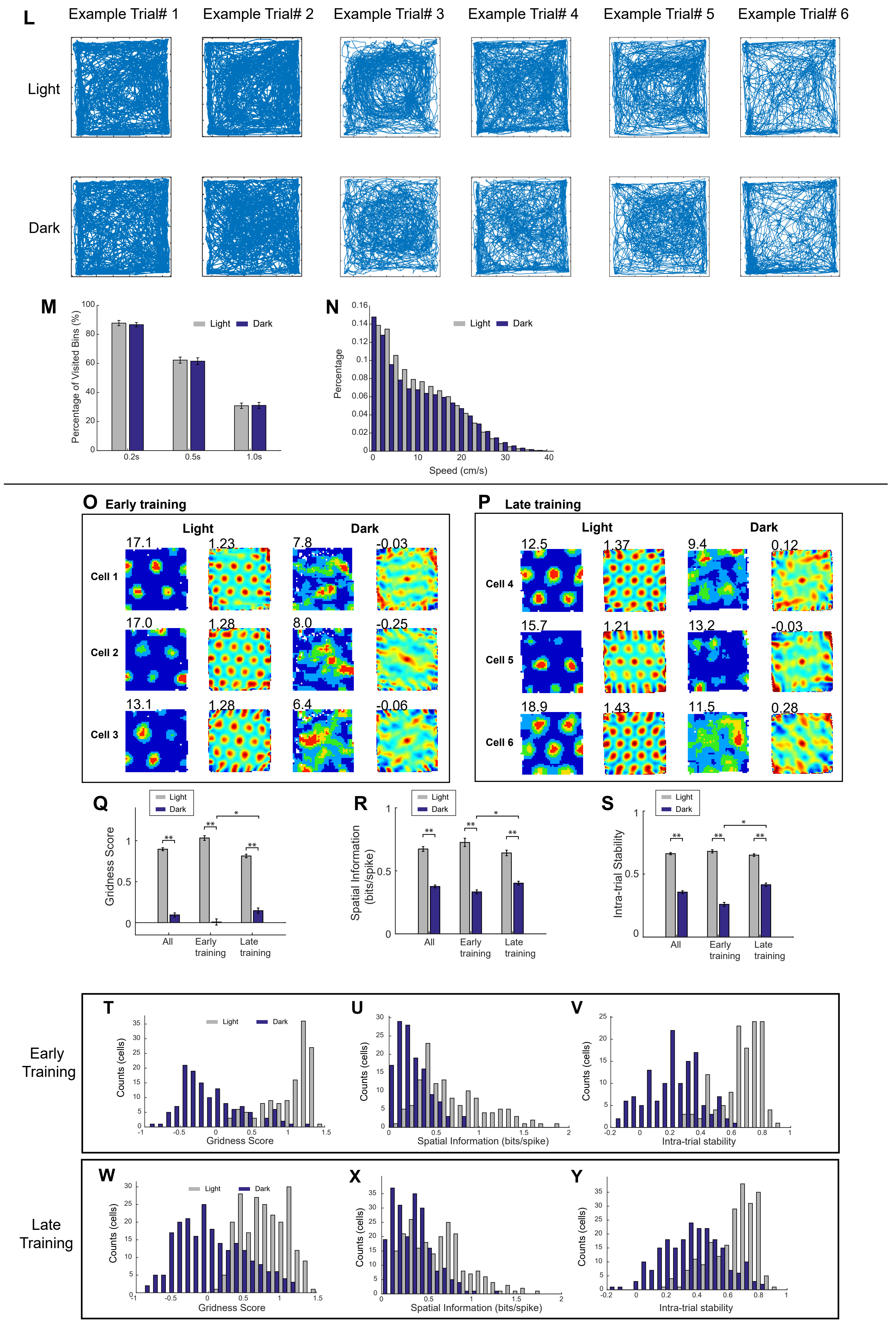
L
Example Trial# 1
Example Trial# 2
Example Trial# 3
Example Trial# 4
Example Trial# 5
Example Trial# 6
Light
Dark
M
Percentage of Visited Bins (%)
Light
Dark
0.2s
0.5s
1.0s
N
Percentage
Light
Dark
Speed (cm/s)
O
Early training
Light
Dark
Cell 1
17.1
1.23
7.8
-0.03
Cell 2
17.0
1.28
8.0
-0.25
Cell 3
13.1
1.28
6.4
-0.06
P
Late training
Light
Dark
Cell 4
12.5
1.37
9.4
0.12
Cell 5
15.7
1.21
13.2
-0.03
Cell 6
18.9
1.43
11.5
0.28
Q
Gridness Score
Light
Dark
All
Early training
Late training
R
Spatial Information (bits/spike)
Light
Dark
All
Early training
Late training
S
Intra-trial Stability
Light
Dark
All
Early training
Late training
Early Training
T
Counts (cells)
Light
Dark
Gridness Score
U
Counts (cells)
Spatial Information (bits/spike)
V
Counts (cells)
Intra-trial stability
Late Training
W
Counts (cells)
Light
Dark
Gridness Score
X
Counts (cells)
Spatial Information (bits/spike)
Y
Counts (cells)
Intra-trial stability

Figure S1, related to main figure 1. Grid cell firing is disrupted by the absence of visual input in the mouse. (A-C) Histograms displaying distributions of Gridness (A), Spatial information (B) and Intra-trial Stability (C) for all Grid cells recorded in the light (grey bars) and dark (blue bars) conditions. Data refers to early training phase (leftmost panels; n trials <4) and late training phase (rightmost panels; n trials >=4). Note that all animals had experienced 3-17 trials in the familiar environment in the light before dark training began. **(D-F) Controlling for possible grid cell resampling over experimental days does not alter results.** Mean values (±SEM) for Gridness (D), Spatial information (E) and Intra-trial Stability (F) for a subset of the complete dataset, derived from only one ensemble of co-recorded grid cells per animal (ensemble with largest number of grid cells, total number of grid cells N=72; light condition in grey, dark condition in blue). Paired t-tests demonstrate significant drops in gridness (t_{71} = 14.75; p <0.001), spatial information (t_{71} = 11.53; p <0.001) and intra-trial stability (t_{71} = 15.13; p <0.001) in the dark, replicating results obtained from analyses of complete dataset and thus excluding the possibility that neuronal resampling results in significant bias. Asterisks denote p <0.001 significance level. **(G-K) A subset of grid cells show spatial firing disruption, despite retaining above-chance gridness scores in the darkness.** (G) Firing rate maps and autocorrelograms of example grid cells with above-chance gridness in the dark (defined as gridness in the dark greater than the 95% confidence threshold used to define neurons as grid cells; see Experimental Procedures and figure S1H). Numbers top left of rate maps show peak firing rate, top left of autocorrelograms show gridness score. Left-most two columns show firing in light, right-most two columns firing in darkness. Six examples from three simultaneously recorded ensembles are shown, illustrating low (top), average (middle) and high (bottom) gridness scores in the dark. See figure S1A for further information regarding where these examples sit in comparison to the population of all gridness scores in the dark. (H) Distribution of the 95% confidence thresholds used to define neurons as grid cells in the light, for all cells defined as grid cells (one threshold per cell, see Experimental Procedures). The mean (± SEM) threshold level was 0.18 (±0.04). All analyses in (I-K) relate to those cells whose gridness score in the dark remained above the 95% confidence threshold derived from that cell's firing in the light (69/277 grid cells). (I) Percentages of grid cells with above-chance gridness in the dark, for data grouped by mouse (left panel) and by experience in darkness (right panel). Black dashed lines show the 95% confidence level for the percentages of above-chance cells expected from spatially random firing in the dark (as spatially random data will score higher than threshold 5% of times). Above-chance gridness in darkness does not appear systematically distributed, with the exception of one mouse (mouse #3) which showed substantial preservation of grid cell firing in darkness. (J) Means and SEMs of gridness (left panel), spatial information (middle panel) and spatial stability (right panel), in light and dark, for grid cells with above-chance gridness in darkness. All three scores show a significant reduction in darkness (paired t-tests): gridness, t(68)=8.66, p<0.001; spatial information, t(68)=9.34, p<0.001; stability, t(68)=13.6, p<0.001. (K) Grid cells with gridness values in the dark which are above or below the 95% confidence level of the shuffled maps do not have significantly different grid scales in the light (t-test: t(275)=0.86, p=0.39). **(L-N) Position and speed sampling are similar in the light and dark conditions.** (L) Twelve representative examples of paths taken by mice in the light (top plots) and related dark (bottom plots) trials, demonstrating even coverage of position across the whole environment in both experimental conditions. (M) Mean values (±SEM) of the percentage of visited bins with occupancy values of at least 0.2 secs (leftmost bars), 0.5 secs (middle set of bars) and 1.0 secs (rightmost bars), during the light (grey bars) and dark (blue) conditions. No differences between dark and light occupancy are evident (paired t-tests, 0.2s occupancy: t(38) = -0.70, p = 0.48; 0.5s occupancy: t(38) = -0.55, p = 0.58; 10s occupancy: t(38) = 0.32, p = 0.74). (N) Histograms displaying distribution of running speed in the light (grey bars) and the dark (blue bars). Overall distributions are similar. Mice on average run slightly faster in the dark (mean speed in light: 10.29cm/s ±0.25; mean speed in dark: 11.13 cm/sec ±0.32; Wilcoxon rank test: p=0.047). **(O-Y) Disruption of grid patterns when visual cues are removed during exploration.** (O-P) Firing rate maps and spatial auto-correlograms for 6 representative grid cells simultaneously recorded in a 60 cm square. Three cells in the right box ('Early Training') were recorded during 2nd exposure to the environment in the dark, 3 cells in the left box ('Late Training'), during 6th exposure. Within boxes, leftmost two columns are from baseline trials in light, and rightmost two columns are from light-dark trials when lights were switched off while animals were exploring the environment (in contrast to the mouse starting exploration in darkness, Figure 1 and S1A-E). Numbers top of firing rate maps are peak firing rate (Hz), those on autocorrelograms are gridness values. (Q-S) Comparisons of firing properties of grid cells between light (grey) and dark trials (blue); gridness (Q), spatial information (R), intra-trial stability (S). Each bar chart shows the mean values (±SEM) for all recorded grid cells (left group of bars), those recorded during days 1-3 of exposure to darkness (middle group of bars) and those recorded during days 4-8 of exposure to darkness (right group of bars). All data were analysed using a 2x2 (light*experience) ANOVA, the resulting statistics are as follows. Gridness: main effect of light $F_{1,346}$=835, p<0.001; experience $F_{1,346}$=1.56, p=0.21; light*experience $F_{1,346}$=37.1, p<0.001. SME experience(dark), p=0.006. Spatial Information: main effect of light $F_{1,346}$=511, p<0.001; experience $F_{1,346}$=0.63, p=0.80; light*experience $F_{1,346}$=30.0, p<0.001. SME experience(dark), p=0.03. Intra-trial stability: main effect of light $F_{1,346}$=847, p<0.001; experience $F_{1,346}$=17.1, p<0.001; light*experience $F_{1,346}$=67.7, p<0.001. SME experience(dark), p=0.03.(T-Y) Histograms displaying distributions of Gridness (T,W), Spatial information (U,X) and Intra-trial Stability (V,Y) for all Grid cells recorded in the light (grey bars) and dark (blue bars) conditions data refers to early training phase (top panels; n trials <4) and late training phase (bottom panels). ** represents significance at p<0.001 level, * represents sigificance at p<0.05.

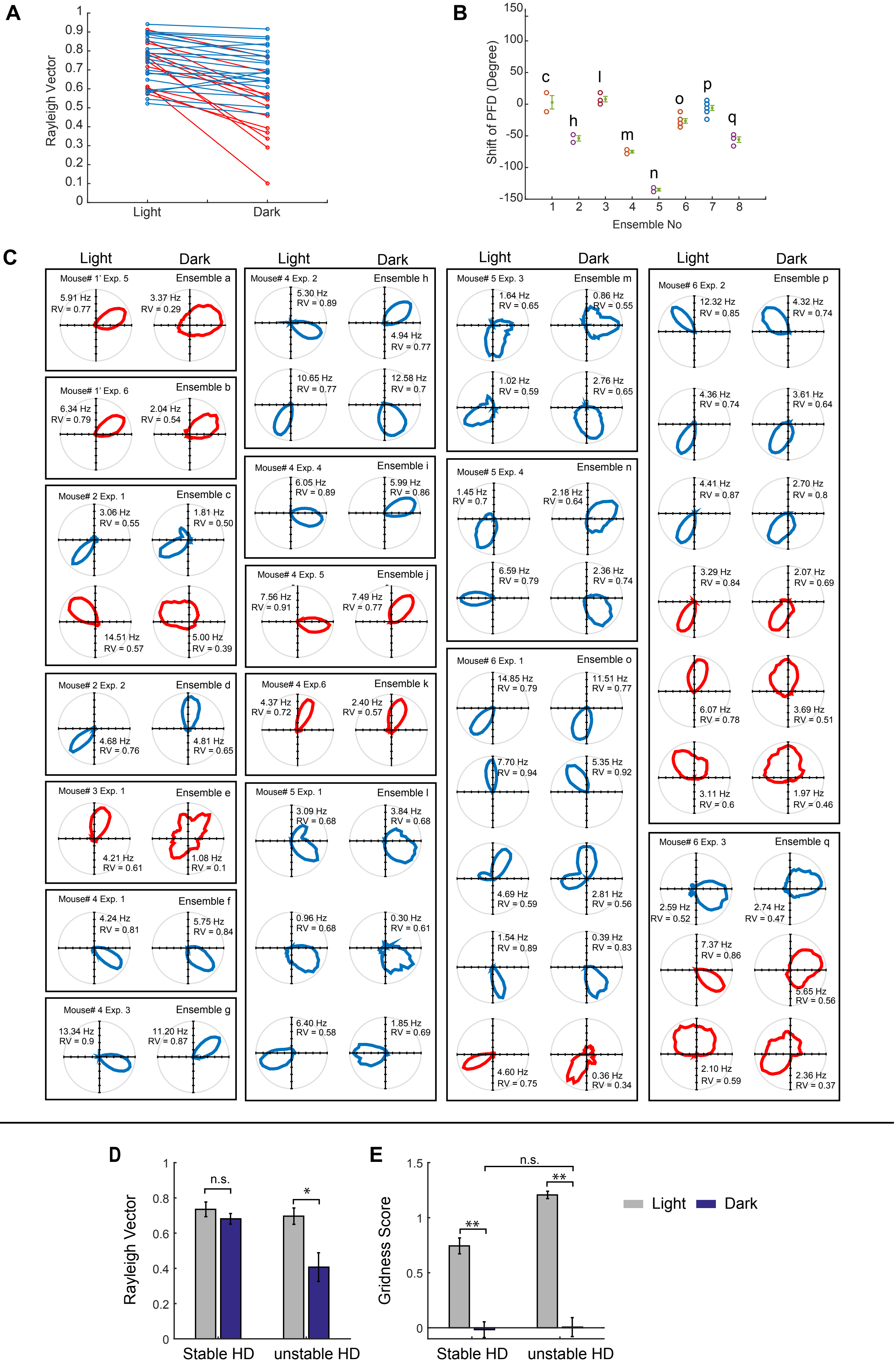
A
Rayleigh Vector
Light
Dark
B
Shift of PFD (Degree)
Ensemble No
c
h
l
m
n
o
p
q
C
Light
Dark
Mouse# 1' Exp. 5
Ensemble a
5.91 Hz RV = 0.77
3.37 Hz RV = 0.29
Mouse# 1' Exp. 6
Ensemble b
6.34 Hz RV = 0.79
2.04 Hz RV = 0.54
Mouse# 2 Exp. 1
Ensemble c
3.06 Hz RV = 0.55
1.81 Hz RV = 0.50
14.51 Hz RV = 0.57
5.00 Hz RV = 0.39
Mouse# 2 Exp. 2
Ensemble d
4.68 Hz RV = 0.76
4.81 Hz RV = 0.65
Mouse# 3 Exp. 1
Ensemble e
4.21 Hz RV = 0.61
1.08 Hz RV = 0.1
Mouse# 4 Exp. 1
Ensemble f
4.24 Hz RV = 0.81
5.75 Hz RV = 0.84
Mouse# 4 Exp. 3
Ensemble g
13.34 Hz RV = 0.9
11.20 Hz RV = 0.87
Mouse# 4 Exp. 2
Ensemble h
5.30 Hz RV = 0.89
4.94 Hz RV = 0.77
10.65 Hz RV = 0.77
12.58 Hz RV = 0.7
Mouse# 4 Exp. 4
Ensemble i
6.05 Hz RV = 0.89
5.99 Hz RV = 0.86
Mouse# 4 Exp. 5
Ensemble j
7.56 Hz RV = 0.91
7.49 Hz RV = 0.77
Mouse# 4 Exp.6
Ensemble k
4.37 Hz RV = 0.72
2.40 Hz RV = 0.57
Mouse# 5 Exp. 1
Ensemble l
3.09 Hz RV = 0.68
3.84 Hz RV = 0.68
0.96 Hz RV = 0.68
0.30 Hz RV = 0.61
6.40 Hz RV = 0.58
1.85 Hz RV = 0.69
Mouse# 5 Exp. 3
Ensemble m
1.64 Hz RV = 0.65
0.86 Hz RV = 0.55
1.02 Hz RV = 0.59
2.76 Hz RV = 0.65
Mouse# 5 Exp. 4
Ensemble n
1.45 Hz RV = 0.7
2.18 Hz RV = 0.64
6.59 Hz RV = 0.79
2.36 Hz RV = 0.74
Mouse# 6 Exp. 1
Ensemble o
14.85 Hz RV = 0.79
11.51 Hz RV = 0.77
7.70 Hz RV = 0.94
5.35 Hz RV = 0.92
4.69 Hz RV = 0.59
2.81 Hz RV = 0.56
1.54 Hz RV = 0.89
0.39 Hz RV = 0.83
4.60 Hz RV = 0.75
0.36 Hz RV = 0.34
Mouse# 6 Exp. 2
Ensemble p
12.32 Hz RV = 0.85
4.32 Hz RV = 0.74
4.36 Hz RV = 0.74
3.61 Hz RV = 0.64
4.41 Hz RV = 0.87
2.70 Hz RV = 0.8
3.29 Hz RV = 0.84
2.07 Hz RV = 0.69
6.07 Hz RV = 0.78
3.69 Hz RV = 0.51
3.11 Hz RV = 0.6
1.97 Hz RV = 0.46
Mouse# 6 Exp. 3
Ensemble q
2.59 Hz RV = 0.52
2.74 Hz RV = 0.47
7.37 Hz RV = 0.86
5.65 Hz RV = 0.56
2.10 Hz RV = 0.59
2.36 Hz RV = 0.37
D
Rayleigh Vector
n.s.
*
Stable HD
unstable HD
E
Gridness Score
n.s.
**
**
Stable HD
unstable HD
Light
Dark

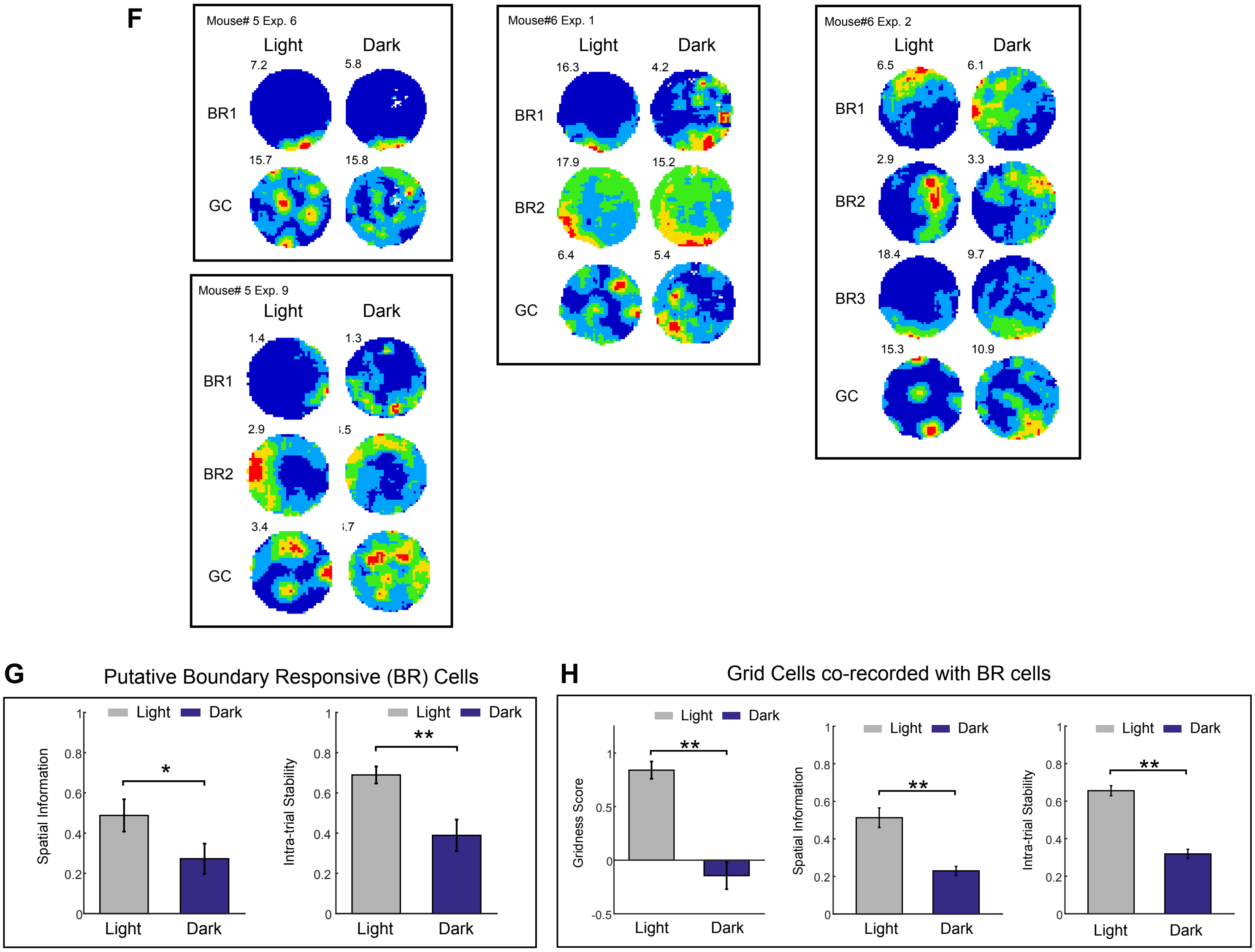

Figure S2, related to main figure 2. (A-C) HD signalling is relatively spared in darkness. (A) Rayleigh Vector (RV) values of all recorded head direction cells (RV>0.5 in light sessions), in both light and dark trials. Lines connect the light and dark values for individual HD cells. Blue lines refer to Stable HD cells ($deltaRV_{light\text{-}dark}$ <=0.12), red to HD cells which become unstable in the dark ($deltaRV_{light\text{-}dark}$ >0.12). (**B**) Co-recorded HD cells retain their relative preferred directions in darkness, regardless of stability. Graph depicting shift in preferred directions between dark and light trials for all HD cells simultaneously recorded with at least another HD cell, either stable or unstable (data refers to all ensembles containing at least 2 HD cells). Data are grouped per ensemble of co-recorded HD cells and letter on top of each vertical group of circles identifies ensemble (related polar plots are displayed in panel S2C). Circles show preferred direction shifts for indivdual HDCs, green lines and error bars show the circular mean and standard deviation of the preferred direction shifts within each ensemble. (C) Polar plots for all Head direction (HD) cells co-recorded with grid cells. For each cell, light trials are shown on the left, dark trials on the right. Black boxes group the 34 individual cells into the 17 simultaneously recorded ensembles from which they are drawn. Number on polar plots are peak firing rate (Hz), and Raleigh Vector (RV) values. Blue polar plots refer to Stable HD cells ($deltaRV_{light\text{-}dark}$ <=0.12), red to HD cells which become unstable in the dark ($deltaRV_{light\text{-}dark}$ >0.12). **(D-E) HD cells' directional tuning is preserved relative to grid firing in the dark, even after controlling for potential resampling of neurons over different experimental days.** Data were analysed from one ensemble per mouse only, that with the greatest number of HD cells (N=15 HD cells, 9 (60%) stable, 6 (30%) unstable). As for the whole dataset, RV values are significantly different between the light and dark condition for unstable HD cells only (panel D; 2x2 ANOVA light*HDC stability, RV: main effect of light, $F_{1,13}$=30.8, p<0.001; HDC stability $F_{1,13}$=6.3, p=0.026; light*HDC stability, $F_{1,13}$=14.5, p=0.002; SME HDC stability(light) p=0.55; SME HDC stability(dark) p=0.03), but co-recorded grid cells (N=49) are equally disrupted whether recorded with stable or unstable HD cells (panel E, 2x2 ANOVA light*HDC stability, gridness: main effect of light, $F_{1,47}$=170, p<0.001; HDC stability $F_{1,47}$=15.0, p<0.001; light*HDC stability, $F_{1,47}$=8.6, p=0.005; SME HDC stability(dark) p=0.84). * denotes significance at p<0.05 level, ** at p<0.001 level. **(F-H) Putative boundary related firing is disrupted in darkness whilst retaining preferential firing near environmental walls.** (F) Firing rate maps for putative boundary responsive cells (BR cells; N=8, top) and examples of co-recorded grid cells (GC cells, bottom) in the light (leftmost maps) and dark (rightmost maps) condition. Black boxes group maps into the 4 simultaneously recorded ensembles from which they are drawn. Numbers top left of firing rate maps indicate peak firing rate (in Hz). (G-H) Comparisons of firing properties of BR cells (G) and grid cells (H) between light (grey) and dark trials (blue). Each bar chart shows the mean values (±SEM) for all recorded BR cells (G) and co-recorded grid cells (H). (G) Both spatial information and intra-trial stability of BR cells are significantly reduced in the dark (paired t-test, spatial information: t(8)=2.84, p=0.022; intra-trial stability: t(8)=4.65, p=0.002). (H) Gridness, spatial information, intra-trial stability of grid cells co-recorded with BR cells all drop significantly in the dark (gridness: t(10)=3.48, p=0.006; spatial information: t(10)=4.61, p=0.001; intra-trial stability: t(10)=4.39, p=0.001).

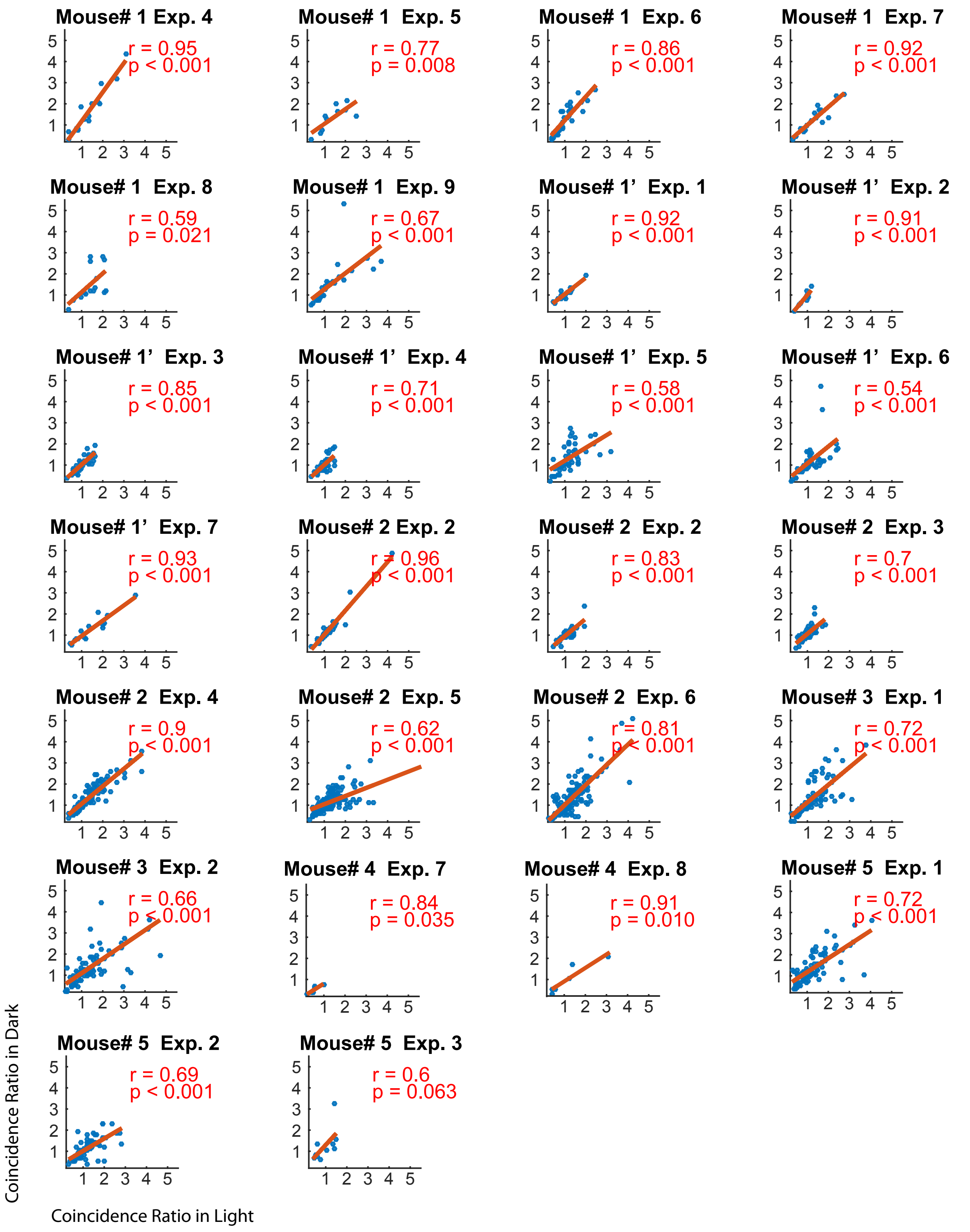

Figure S3, related to main figure 3. Grid cells retain temporal coupling at short timescales (1.5sec) in darkness. Coincidence ratios of temporal cross-correlograms between grid cells pairs in light and darkness, split into simultaneously recorded ensembles. Each plot shows the coincidence ratio of all pairings between a set of simultaneously recorded grid cells, in darkness in comparison to that in light. Only ensembles in which 4 or more grid cells were simultaneously recorded are shown. Numbers in red show the r and p-values for linear regression between the light and dark coincidence ratios.

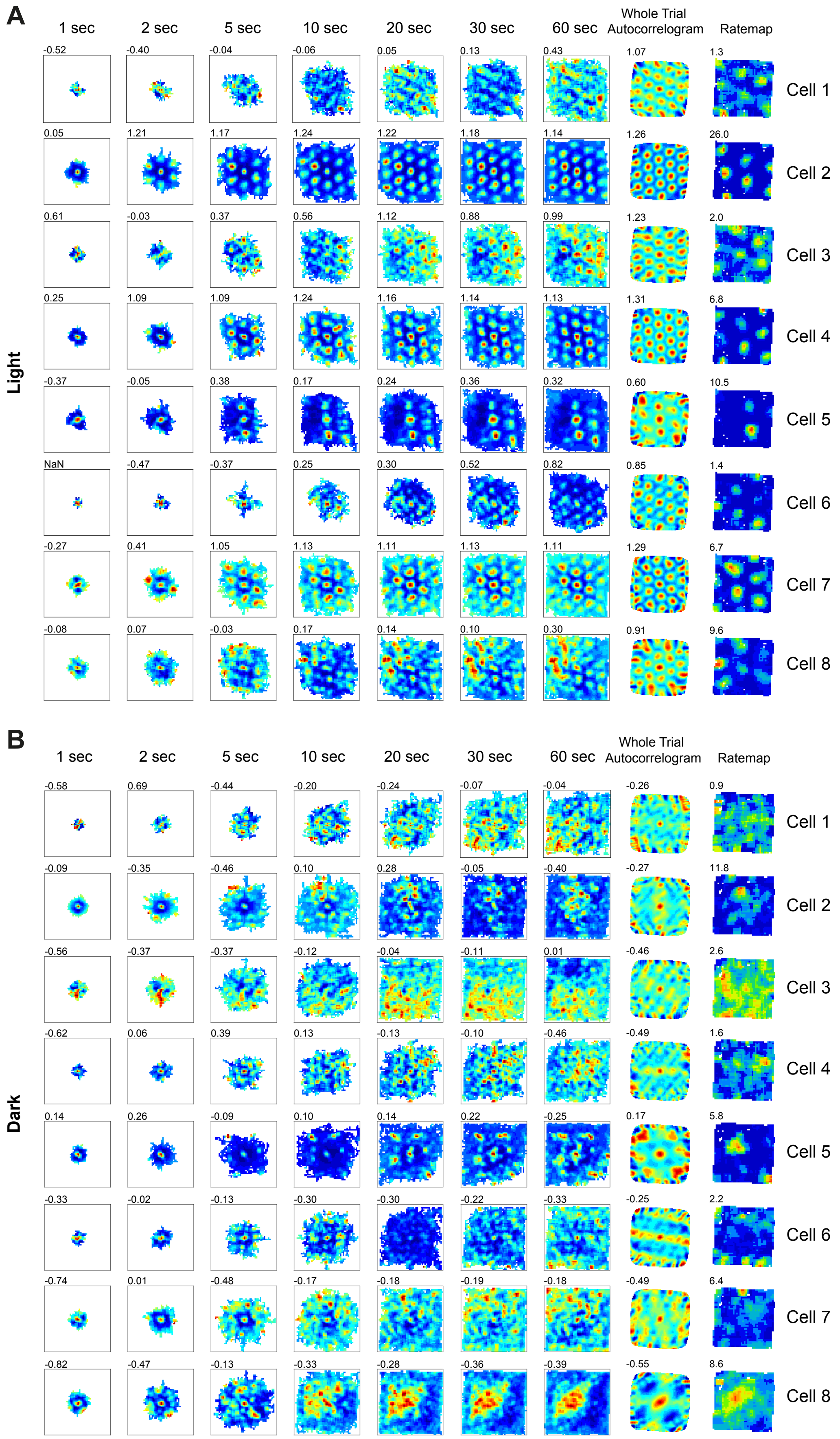

Figure S4, realted to main figure 4. No evidence for hexagonal symmetry in grid cell firing, even at short timescales. (A) 2D Time-windowed spatial displacement firing rate maps (1, 2, 5, 10, 30 and 60 sec windows; five leftmost columns), for a representative example of co-recorded grid cell ensemble in light, and respective whole trial spatial auto-correlograms (the sixth column) and firing rate maps (seventh column) (B) As for (A), the same set of cells recorded in darkness (same ensemble from which the 3 representative examples shown in Figure 4 were drawn).

Supplemental Experimental Procedures

Subjects and surgery. Subjects (6 C57Bl/6 mice) were aged 2-5 months and weighed 23-30 grams at the time of surgery. Mice were housed under 12:12 inverted light-dark cycle, with lights on at 10am. All work was carried out under the Animals (Scientific Procedures) Act 1986 and according to Home Office and institutional guidelines. Mice were implanted with custom-made microdrives targeting the mEC. Each microdrive was loaded with 32 HML-coated 17-μm Platinum-Iridium (90-10%) electrodes twisted into 8 separate tetrodes. Electrodes were electroplated in a Platinum solution to <200kΩ impedance. Following surgery animals were allowed at least one week recovery.

Behavioural Training/Recording environment details. After recovery from surgery, mice were exposed to the same recording arena every day (20 mins day) to screen for grid cell activity. Tetrodes were lowered by 62.5 um each day, until grid cell activity was identified, after which recording sessions began (and tetrodes were no longer moved after this time point). One of three different arenas was used for different mice: (1) a 50 cm diameter circle, (2) a 60 cm diameter circle, (3) a 60x60 cm square. All recording arenas were placed on a black Trespa 'Toplab' surface (Trespa International B.V., Weert, Netherlands) that served as a floor, and surrounded by a circular set of black curtains. A white cue-card (A0, 84 x 119 cm), illuminated by a 40 W lamp, was the only directionally polarising cue within the black curtains. One mouse (mouse #1) underwent the process of being familiarised with the recording arena and then exposed to darkness twice, the second time in a different laboratory and using a different recording arena to those used the first time. Data from exposure in the second laboratory was treated separately to that from the first when quantifying experience, data from mouse #1 that was collected in the second laboratory is designated mouse #1'.

Experimental Protocol. Extra-cellular waveforms were recorded using the "DACQ" recording system (Axona). During recording, the mouse's position and head orientation was tracked (50Hz sampling rate) using two infra-red LEDs attached to the micro-drive at a fixed angle and spacing (5 cm apart). Brief losses of LED data due to cable obstruction were corrected with linear interpolation between known position values. Interpolation was carried out for each LED separately. The position values for each LED were then smoothed, separately, using a 400ms long boxcar filter. Finally, the separate LED positions were used to calculate the averaged position and heading for each position sample. During 'dark' trials, the lights were turned off before the mouse entered into the recording arena, and were not turned on again until he had been removed from it. 'Dark' trials were conducted in complete darkness: all sources of visible light in the laboratory were eliminated and the experimenter used infra-red night vision goggles to conduct the experiments. In a sub-set of experiments, 'light-dark' trials were conducted: in these trials, mice were placed in the arena with the lights turned on, after 10 mins the lights were turned off, and recording continued for a further 20 mins (see Figure S1O-Y).

Rate maps and gridness score. Spike sorting was performed offline using an automated clustering algorithm (KlustaKwik [S1]) followed by a manual review and editing step using an interactive graphical tool (waveform, http://d1manson.github.io/waveform/). After spike sorting, firing rate maps were constructed by binning animals' positions into 1.5 x 1.5cm bins, assigning spikes to each bin, smoothing both position maps and spike maps separately using a 5x5 boxcar filter, and finally dividing the smoothed spike maps by the smoothed position maps. Then spatial autocorrelograms were calculated from the rate maps according to [S2]:

$$r(\tau_x,\tau_y) = \frac{n\sum\lambda(x,y)\lambda(x-\tau_x,\ y-\tau_y) - \sum\lambda(x,y)\sum\lambda(x-\tau_x,\ y-\tau_y)}{\sqrt{n\sum\lambda(x,y)^2 - (\sum\lambda(x,y))^2}.\sqrt{n\sum\lambda(x-\tau_x,\ y-\tau_y)^2 - (\sum\lambda(x-\tau_x,\ y-\tau_y))^2}}$$

Where $r(\tau_x,\tau_y)$is the autocorrelation between bins with spatial offset of τ_x and τ_y. $\lambda(x,y)$ is firing rate in bin (x,y) and n is the number of bins. Six closest peaks of the autocorrelogram were defined by finding six local maxima, closest to (but excluding) the central peak. Gridness was calculated by defining a mask on the spatial autocorrelogram centred on the central peak, but excluding the peak itself (from the centre to the half-height), bounded by a circle with the distance from the central peak to the furthest peak of the six closest peaks. The masked area was rotated in 30° increments up to 150°, and for each rotation the Pearson product moment correlation coefficient was calculated against the un-rotated mask. Gridness was then expressed as the lowest correlation obtained for rotations of 60° and 120° minus the highest correlation obtained at 30°, 90° or 150°.

Grid cell inclusion criterion. Cells were classified as grid cells if their gridness score in 'light' trial exceeded the 95th percentile of a distribution of 1000 gridness scores derived by spatially shuffling the spike data for that cell. To spatially shuffle data for each cell, a fixed time offset was added to all spike times for that cell, and those spike times which then occurred after the end of the trial were 'wrapped' onto the beginning of the trial, by subtracting from their times the trial duration. Position data was unchanged. In this way, the temporal dynamics of the spike train were preserved, but the relation of the spikes to position was uncoupled. Following this procedure, rate maps were constructed and gridness scores calculated as described above. To generate a population of 1000 spatially shuffled gridness scores for each cell, the above procedure was repeated using a set of 1000 offsets, evenly spaced ranging from 1s to trial duration minus 1s.

Measures of stability and spatial tuning. *Intra-trial stability* was measured by correlating the firing rates of spatially corresponding bins from the first and the second halves of a trial, using only those bins in which firing rate > 0 Hz in both halves of the trial.

Spatial information was calculated following [S3]. The estimate of the mutual information $I(R|X)$ between firing rate R and location X is:

$$I(R|X) = \sum_{i} p(\vec{x_i}) f(\vec{x_i}) \log_2(\frac{f(\vec{x_i})}{F})$$

where $p(\vec{x_i})$ is the probability for the animal being at location (or facing direction) $\vec{x_i}$, $f(\vec{x_i})$ is the firing rate observed at $\vec{x_i}$, and F is the overall firing rate of the cell. $I(R|X)$ was then divided by the overall mean firing rate of the cell in the trial, giving an estimate in bits/spike.

Rayleigh vector (head direction cells). Directional firing rate maps were constructed analogously for those of 2-dimensions, using 6° bins and a 30° wide boxcar smoothing filter. The directional tuning of the cell was measured using the length of the mean resultant vector (Rayleigh Vector; RV) of the bins of directional firing rate map. A cell was defined as a head direction (HD) cell if it displayed an RV ≥ 0.5 in the 'light' trial. HD cells were classified as 'stable dark' HD cells (SD-HD cells) if their RV in the dark trial was reduced by an amount less than or equal to the overall standard deviation of the RV scores in the light (≤0.12).

Speed modulation of theta frequency and firing rate. *Theta frequency.* After band-pass filtering (between 5 and 11Hz), the Hilbert transform was used to define an instantaneous phase for each data point in the filtered LFP signal, and instantaneous phases were 'unwrapped', such that there were no sudden jumps between 0 and 2 π. Instantaneous frequency for LFP sample N was then defined as (phase(N)-phase(N-1))/(2π*sample_rate). The theta frequency for each position sample (20ms duration) was then calculated as the mean of the instantaneous frequencies from the corresponding time points. Position samples were then sorted on the basis of running speed (5cm/s bins running between 5cm/s and 35cm/s), and the overall mean frequency for each speed bin calculated.

Firing rate. First, speed-modulated grid cells were classified from the general population of grid cells following [S4]. Briefly, the degree of speed modulation for each grid cell was characterised by first defining the instantaneous firing rate of the cell as the number of spikes occurring in each position bin divided by the sampling rate. This estimate of instantaneous rate was then smoothed using a 400ms long boxcar filter (matching that applied to position, see above). Finally, a linear correlation was computed between the running speeds and firing rates across all position samples in a trial, and the resulting r-value was taken to characterise the degree of speed modulation for the cell. To be defined as speed-modulated, the r-value for a grid cell had to exceed the 99th percentile of a population of r-values derived by spatially shuffling the spike train. Spatial shuffling was performed in the same way as for calculation of gridness, see 'Grid cell inclusion criterion, above. After speed-modulated grid cells were defined (those grid cells speed-modulated in either the light, or the dark, or both), the nature of speed modulation in the light or the dark was described by sorting position samples on the basis of running

speed, and calculating the mean firing rate, for each cell and for each speed bin, based on the smoothed estimates of instantaneous firing described above.

Coincidence ratio and temporal cross-correlograms. For each pair of simultaneously recorded grid cells, A and B, we identified the sets of spikes from cell B falling within ±2s of each spike in cell A. Then, by subtracting the time of the relevant A spike, the collection of times from cell B were expressed as deltas. The temporal cross-correlograms shown in Figure 3 display this list of deltas as a histogram with 50ms bins, with the counts normalised by the total number of spikes from cell A and by the bin width. Thus, the y-axis values give the mean, over all spikes from cell A, of the rate of cell B at the given temporal offset from a spike in cell A.

To compute the "coincidence-ratio" from the temporal autocorrelogram, we took the mean of the section with $-0.5s < \Delta t < +0.5s$, and divided by the mean of the two sections with $-1.5s < \Delta t < -1s$ and $1s < \Delta t < 1.5s$. The preservation of coincident/non-coincident firing relationships in the dark was then tested using linear regression between the light and dark ratios.

Time-windowed spatial displacement firing rate maps. A time-windowed spatial displacement firing rate map was used to assess short-term spatial structure in grid cell firing in the dark. For each spike, the 2-dimensional displacements of other spikes fired, and positions occupied, within T seconds were counted, and the histograms of displacement were formed for all spikes (bin size 2.4x2.4cm) and smoothed (3x3 boxcar). The histograms for spikes and positions were divided to estimate the probability of spike pairs per second as a function of their displacement. Displacements represented by fewer than 2s of occupancy were not shown. Gridness of time-windowed auto-correlograms was assessed using the gridness measure described above, with the exception that, the six closest peaks were not defined, rather, the gridness mask derived from the whole trial auto-correlogram was used instead. This was found to increase the gridness scores of time-windowed auto-correlograms, as the six closest peaks were not clearly defined in all cases. 1-dimensional distance time-windowed maps were constructed exactly as for the 2D maps described above, but x and y values were collapsed to Pythagorean distance before constructing a 1D rate map, which was smoothed with a 3 bin long boxcar kernel. Exactly as for 2D maps, the firing rate of the real data was re-expressed as standard deviations above the mean firing rate of a set of 100 time-windowed firing rate maps derived from spatially-shuffled data (spatial shuffling performed exactly as described above under "Grid cell inclusion criterion"). This normalisation was performed separately for each spatial bin in the rate map. Lastly, the distance scale of time-windowed distance maps was normalised such that a distance of 1 was equal to the mean grid cell wavelength in the light, for that ensemble of co-recorded cells (as all ensembles consisted of one grid module only, averaging across the ensemble provided a more reliable estimate of grid wavelength). For any given cell, the same normalisation factor was applied to light and dark time-windowed maps.

References

1. Kadir, S. N., Goodman, D. F. M., and Harris, K. D. (2014). High-dimensional cluster analysis with the masked EM algorithm. Neural Comput. *26*, 2379–94.

2. Sargolini, F., Fyhn, M., Hafting, T., McNaughton, B. L., Witter, M. P., Moser, M. B., and Moser, E. I. (2006). Conjunctive representation of position, direction, and velocity in entorhinal cortex. Science (80-.). *312*, 758–762.

3. Skaggs, W. E., McNaughton, B. L., Gothard, K. M., and Markus, E. J. (1993). An information-theoretic approach to deciphering the hippocampal code. Adv Neural Inf Process Syst *5*, 1030–1037.

4. Kropff, E., Carmichael, J. E., Moser, M.-B., and Moser, E. I. (2015). Speed cells in the medial entorhinal cortex. Nature *523*, 419–24.
